# Supplementary material for: Abundance and diversity of host-seeking adult female mosquitoes in a coastal ecosystem in southern Mexico
Source: PLoS Negl Trop Dis. 2025 Jun 9;19(6):e0012316. doi: 10.1371/journal.pntd.0012316 (PMC12173415; doi:10.1371/journal.pntd.0012316)
Supplement: S1 Table — The table shows key metrics including sample size (T), total number of incidences (U), observed species richness (S.obs), estimated sample coverage (SC), and the first ten incidence frequency counts (Q1-Q10). Data are categorized by sampling season (Norte, Dry, Rainy) and sampling hour (01:00, 05:00, 09:00, 13:00, 17:00, 21:00). (DOCX) [file pntd.0012316.s001.docx]

|  | T | U | S.obs | SC | Q1 | Q2 | Q3 | Q4 | Q5 | Q6 | Q7 | Q8 | Q9 | Q10 |
| --- | --- | --- | --- | --- | --- | --- | --- | --- | --- | --- | --- | --- | --- | --- |
| **Sampling season** |  |  |  |  |  |  |  |  |  |  |  |  |  |  |
| Norte | 6 | 100 | 28 | 0.947 | 6 | 2 | 5 | 6 | 3 | 6 | 0 | 0 | 0 | 0 |
| Dry | 6 | 18 | 8 | 0.815 | 4 | 2 | 0 | 1 | 0 | 1 | 0 | 0 | 0 | 0 |
| Rainy | 6 | 67 | 20 | 0.921 | 6 | 2 | 4 | 0 | 3 | 5 | 0 | 0 | 0 | 0 |
| **Sampling hour** |  |  |  |  |  |  |  |  |  |  |  |  |  |  |
| 01:00 | 3 | 36 | 21 | 0.875 | 9 | 9 | 3 | 0 | 0 | 0 | 0 | 0 | 0 | 0 |
| 05:00 | 3 | 38 | 23 | 0.841 | 11 | 9 | 3 | 0 | 0 | 0 | 0 | 0 | 0 | 0 |
| 09:00 | 3 | 21 | 13 | 0.857 | 6 | 6 | 1 | 0 | 0 | 0 | 0 | 0 | 0 | 0 |
| 13:00 | 3 | 22 | 13 | 0.905 | 5 | 7 | 1 | 0 | 0 | 0 | 0 | 0 | 0 | 0 |
| 17:00 | 3 | 29 | 19 | 0.755 | 11 | 6 | 2 | 0 | 0 | 0 | 0 | 0 | 0 | 0 |
| 21:00 | 3 | 39 | 25 | 0.812 | 13 | 10 | 2 | 0 | 0 | 0 | 0 | 0 | 0 | 0 |

**S1 Table. Sample coverage estimates derived from host-seeking female mosquito incidence data across different sampling seasons and hours.**

T = Sample size; U = Total number of incidences; S.obs = Observed species richness; SC = Estimated sample coverage; Q1-Q10 = the first ten counts of incidence frequency.
