# Supplementary material for: Abundance and diversity of host-seeking adult female mosquitoes in a coastal ecosystem in southern Mexico
Source: PLoS Negl Trop Dis. 2025 Jun 9;19(6):e0012316. doi: 10.1371/journal.pntd.0012316 (PMC12173415; doi:10.1371/journal.pntd.0012316)
Supplement: S2 Table — The table presents the estimated effects of vegetation type, season, and sampling hour on mosquito abundance for the genera Aedes, Psorophora, Mansonia, Culex, and Anopheles. Vegetation types include semideciduous forest modified by buildings (BLDSM), dominant semideciduous forest & mangrove (SMMG), and dominant mangrove & semideciduous forest (MGSM). Results are shown as estimates with standard errors, Wald χ² statistics, and corresponding p-values. (DOCX) [file pntd.0012316.s002.docx]

**S2 Table.** **Estimated effects of environmental covariates on the abundance of host-seeking female mosquitoes for the top five most abundant genera in the study area.**

|  | Estimate | Standard error | Wald χ^2^ | *p*-value |
| --- | --- | --- | --- | --- |
|  | ***Aedes*** | | | |
| **Vegetation:** SMMG (Ref: BLDSM) | 0.076 | 0.073 | 1.100 | 0.294 |
| MGSM (Ref: BLDSM) | -0.206 | 0.104 | 3.900 | 0.048 |
| **Season:** Dry (Ref: Norte) | 0.644 | 0.228 | 7.940 | 0.004 |
| Rainy (Ref: Norte) | 0.031 | 0.228 | 0.020 | 0.890 |
| **Hour:** 05:00 (Ref: 01:00) | -0.262 | 0.151 | 3.020 | 0.082 |
| 09:00 (Ref: 01:00) | 0.015 | 0.323 | 0.000 | 0.961 |
| 13:00 (Ref: 01:00) | 0.287 | 0.202 | 2.020 | 0.154 |
| 17:00 (Ref: 01:00) | -0.138 | 0.372 | 0.140 | 0.709 |
| 21:00 (Ref: 01:00) | -0.171 | 0.060 | 7.910 | 0.004 |
|  | ***Psorophora*** | | | |
| **Vegetation:** SMMG (Ref: BLDSM) | 1.198 | 0.282 | 18.060 | < 0.0001 |
| MGSM (Ref: BLDSM) | 0.611 | 0.236 | 6.680 | 0.009 |
| **Season:** Dry (Ref: Norte) | -41.324 | 1.162 | 1264.000 | < 0.0001 |
| Rainy (Ref: Norte) | -0.029 | 0.592 | 0.000 | 0.960 |
| **Hour:** 05:00 (Ref: 01:00) | 0.535 | 0.803 | 0.440 | 0.506 |
| 09:00 (Ref: 01:00) | 1.008 | 0.221 | 20.800 | < 0.0001 |
| 13:00 (Ref: 01:00) | 2.320 | 0.367 | 39.900 | < 0.0001 |
| 17:00 (Ref: 01:00) | 1.638 | 0.205 | 63.640 | < 0.0001 |
| 21:00 (Ref: 01:00) | 0.758 | 0.810 | 0.880 | 0.350 |
|  | ***Mansonia*** | | | |
| **Vegetation:** SMMG (Ref: BLDSM) | 0.809 | 0.160 | 25.430 | < 0.0001 |
| MGSM (Ref: BLDSM) | 1.324 | 0.983 | 1.810 | 0.180 |
| **Season:** Dry (Ref: Norte) | -3.149 | 0.653 | 23.260 | < 0.0001 |
| Rainy (Ref: Norte) | 0.612 | 0.653 | 0.880 | 0.350 |
| **Hour:** 05:00 (Ref: 01:00) | 0.047 | 0.475 | 0.010 | 0.919 |
| 09:00 (Ref: 01:00) | -0.758 | 0.762 | 0.990 | 0.319 |
| 13:00 (Ref: 01:00) | 0.831 | 0.228 | 13.340 | 0.0002 |
| 17:00 (Ref: 01:00) | 1.500 | 0.797 | 3.550 | 0.059 |
| 21:00 (Ref: 01:00) | -7.31e-17 | 0.199 | 0.000 | 1.000 |
|  | ***Culex*** | | | |
| **Vegetation:** SMMG (Ref: BLDSM) | -0.738 | 0.451 | 2.690 | 0.100 |
| MGSM (Ref: BLDSM) | 0.788 | 0.627 | 1.580 | 0.210 |
| **Season:** Dry (Ref: Norte) | -1.005 | 0.398 | 6.380 | 0.012 |
| Rainy (Ref: Norte) | -0.369 | 0.398 | 0.860 | 0.354 |
| **Hour:** 05:00 (Ref: 01:00) | 0.451 | 0.544 | 0.690 | 0.406 |
| 09:00 (Ref: 01:00) | -3.469 | 1.018 | 11.620 | < 0.001 |
| 13:00 (Ref: 01:00) | -3.469 | 0.990 | 12.270 | < 0.001 |
| 17:00 (Ref: 01:00) | -0.414 | 0.825 | 0.250 | 0.615 |
| 21:00 (Ref: 01:00) | -0.777 | 0.408 | 3.630 | 0.056 |
|  | ***Anopheles*** | | | |
| **Vegetation:** SMMG (Ref: BLDSM) | -0.664 | 0.229 | 8.430 | 0.003 |
| MGSM (Ref: BLDSM) | 0.495 | 0.152 | 10.540 | 0.001 |
| **Season:** Dry (Ref: Norte) | -1.877 | 0.424 | 19.600 | < 0.0001 |
| Rainy (Ref: Norte) | -1.877 | 0.424 | 19.600 | < 0.0001 |
| **Hour:** 05:00 (Ref: 01:00) | -0.090 | 0.175 | 0.260 | 0.607 |
| 09:00 (Ref: 01:00) | -1.11e+08 | 3.66e+06 | 923.290 | < 0.0001 |
| 13:00 (Ref: 01:00) | -3.830 | 0.692 | 30.670 | < 0.0001 |
| 17:00 (Ref: 01:00) | -0.486 | 0.380 | 1.630 | 0.201 |
| 21:00 (Ref: 01:00) | -0.715 | 0.199 | 12.930 | < 0.001 |
