# Supplementary material for: Abundance and diversity of host-seeking adult female mosquitoes in a coastal ecosystem in southern Mexico
Source: PLoS Negl Trop Dis. 2025 Jun 9;19(6):e0012316. doi: 10.1371/journal.pntd.0012316 (PMC12173415; doi:10.1371/journal.pntd.0012316)
Supplement: S1 Appendix — Each entry includes the mosquito species, the pathogen it transmits, and the corresponding reference with its DOI or URL for further reading. (PDF) [file pntd.0012316.s003.pdf]

Species	doi/url	Reference
An. crucians	10.4269/ajtmh.1936.s1-16.159<br>10.1146/annurev.mi.14.100160.001401<br>https://pubmed.ncbi.nlm.nih.gov/8827602/<br>10.1603/0022-2585(2007)44[117:wnviim]2.0.co;2<br>https://pubmed.ncbi.nlm.nih.gov/19706914/<br>10.1603/me13049<br>10.1186/s13071-019-3874-0	Boyd MF, Kitchen SF, Mulrennan JA. On the relative susceptibility of the inland and coastal varieties of An. crucians Wied. To P. falciparum Welch. Am J Trop Med 1936; 16:159–161.<br>Kissling RE. The arthropod-borne viruses of man and other animals. Annu Rev Microbiol 1960; 14:261–282<br>Mitchell CJ, Morris CD, Smith GC, Karabastos N, et al. Arboviruses associated with mosquitoes from nine Florida counties during 1993. J Am Mosq Control Assoc 1996;12:255-262.<br>Cupp EW, Hassan HK, Yue X, Oldland WK, Lilley BM, Unnasch TR. West Nile virus infection in mosquitoes in the mid-south USA, 2002-2005. J Med Entomol 2007;44:117-125.<br>Cohen SB, Lewoczko K, Huddleston DB, Moody E, et al. Host feeding patterns of potencial vectors of Eastern equine encephalitis virus at an epizootic focus in Tenessee. Am J Trop Med Hyg 2009;81:452-456.<br>Turell MJ, Britch SC, Aldridge RL, Kline DL, et al. Potential for mosquitoes (Diptera: Culicidae) from Florida transmit Rift Valley fever virus. J Med Entomol 2013;50:1111-1117.<br>Spence Beaulieu MR, Federico JL, Reiskind MH. Mosquito diversity and dog heartworm prevalence in suburban areas. Parasit Vectors 2020;13:1-12.
An. vestittipennis	10.1016/0035-9203(91)90010-v	Loyola EJ, Arredondo JI, Rodriguez MH, Brown DN, Vaca-Marin MA. Anopheles vestitipennis, the probable vector of Plasmodium vivax in the Lacandon fores of Chiapas, Mexico. Trans R Soc Med Hyg 1991;85:171-174
An. albimanus	10.7705/biomedica.v5i1-2.1895<br>10.1186/s13071-019-3331-0<br>https://www.cabdirect.org/cabdirect/abstract/19691000052	Olano VA, Carrillo MP, Espinal CA. Estudios de infectividad de la especie Anopheles albimanus Wiedemann, 1820 (Diptera: Culicidae) cepa Cartagena, con plasmodios humanos. Biomedica 1985;5:5-10.<br>González-Cerón L, Rodríguez MH, Nettel-Cruz JA, Hernández-Ávila JE, et al. Plasmodium vivax CSP-Pvs25 variants from southern Mexico produce distinct patterns of infectivity for Anopheles albimanus versus An. pseudopunctipennis, in each case independent of geographical origin. Parasit Vectors 2019;12:86.<br>Collins, W.; Harrison, A. Studies of Tensaw Virus in Anopheles quadrimaculatus, A. albimanus, and A. maculatus. Mosq. News 1967, 27, 1–5.
Ae. squamipennis	https://horizon.documentation.ird.fr/exl-doc/pleins_textes/pleins_textes_6/b_fdi_33-34/38274.pdf<br>10.1111/j.1365-294X.2008.03764.x<br>https://pubmed.ncbi.nlm.nih.gov/2863989/	Degallier N, Travassos da Rosa APA, Vasconcelos PFC, Herve JP, Sa Filho GC, Travassos da Rosa ES, Travassos da Rosa JFS, Rodrigues SG. Modifications of arbovirus transmission in relation to construction of dams in Brazilia Amazonia. Public health in the amazon 1992; 44:124-135.<br>Gager AB, Loaiza J, Dearborns DC, Berminham E. Do mosquitoes filter the acces of Plasmodium cytocrome b lineages to an avian host. Molecular ecology 2008; 17:2552-2561<br>Mitchell, C.J., T.P. Monath, M.S. Sabattini, C.B. Cropp, J.F. Daffner, C.H. Calisher, W.L. Jakob & H.A. Christensen. Arbovirus investigations in Argentina, 1977–1980 II. Arthropod collection and virus isolations from argentine mosquitoes. The American Journal of Tropical Medicine and Hygiene 1985; 34(5): 945–955.
Ae. angustivittatus	10.4269/ajtmh.1963.12.924<br>10.1093/oxfordjournals.aje.a120870	Peña A, Kumm HW. Algunas consideraciones generals sobre la miasis cutanea (torsalo) en Costa Rica. Rev Med 1939; 64:635–641.<br>de Rodaniche E, Galindo P. Ecological observations of Ilheus virus in vicinity of Almirante, Republic of Panama. Am J Trop Hyg 1963; 12:924–928<br>Grayson MA, Galindo P. Epidemiologic studies of Venezuelan equine encephalitis virus in Almirante, Panama. Am J Epidemiol 1968; 88:80–96.<br>Alvarado-Torres H., Viveros-Santos V., Torres-Monzón J.A., López-Ordóñez T., Torres-Chable O.M., Casas-Martínez M. Detección de Dirofilaria immitis (Spiruridae: Onchocercidae) en la comunidad de mosquitos (Diptera: Culicidae) de cementerios de la región Soconusco, Sur de México. 2019 Entomol Mex: 6; 480-486
Ae. fulvus	https://horizon.documentation.ird.fr/exl-doc/pleins_textes/pleins_textes_6/b_fdi_33-34/38274.pdf<br>10.1603/0022-2585(2005)042[0891:IOVFMD]2.0.CO;2	Degallier N, Travassos da Rosa APA, Vasconcelos PFC, Herve JP, Sa Filho GC, Travassos da Rosa ES, Travassos da Rosa JFS, Rodrigues SG. Modifications of arbovirus transmission in relation to construction of dams in Brazilia Amazonia. Public health in the amazon 1992; 44:124-135.<br>Turell MJ, O'Guinn ML, Jones JW, Sardelis MR, Dohm DJ, Watts DM, Fernandez R, Travassos da Rosa A, Guzman H, Tesh R, Rossi CA, Ludwig V, Mangiafico JA, Kondig J, Wasieloski LP Jr, Pecor J, Zyzak M, Schoeler G, Mores CN, Calampa C, Lee JS, Klein TA. Isolation of viruses from mosquitoes (Diptera: Culicidae) collected in the Amazon Basin region of Peru. J Med Entomol. 2005 Sep;42(5):891-8.
	https://pubmed.ncbi.nlm.nih.gov/13724163/<br>10.1146/annurev.mi.14.100160.001401	Downs WG, Spence L, Aitken TH, Whitman LE. Cache Valley virus, isolated from a Trinidadian mosquito, Aedes scapularis. West Indian Med J. 1961; 10: 13–15. PMID: 13724163<br>Kissling RE. The arthropod-borne viruses of man and other animals. Annu Rev Microbiol 1960; 14:261–282

Ae. scapularis	https://horizon.documentation.ird.fr/exl-doc/pleins_textes/pleins_textes_6/b_fdi_33-34/38274.pdf	Degallier N, Travassos da Rosa APA, Vasconcelos PFC, Herve JP, Sa Filho GC, Travassos da Rosa ES, Travassos da Rosa JFS, Rodrigues SG. Modifications of arbovirus transmission in relation to construction of dams in Brazilia Amazonia. Public health in the amazon 1992; 44:124-135.
		Arnell, J. H. 1976. Mosquitoes studies (Diptera, Culicidae) XXXIII A revision of the Scapularis group of Aedes (Ochlerotatus). Contributions of the American Entomological Institute 13 (3): 1-144
	10.1111/j.1365-2915.2010.00884.x	Manrique-Saide P, Escobedo-Ortegón J, Bolio-González M, Sauri-Arceo C, Dzib-Florez S, Guillermo-May G, Ceh-Pavía E, Lenhart A. Incrimination of the mosquito, Aedes taeniorhynchus, as the primary vector of heartworm, Dirofilaria immitis, in coastal Yucatan, Mexico. Med Vet Entomol. 2010 Dec;24(4):456-60.
	10.1371/journal.pntd.0009494	Elbadry MA, Durães-Carvalho R, Blohm GM, Stephenson CJ, Loeb JC, et al. (2021) Orthobunyaviruses in the Caribbean: Melao and Oropouche virus infections in school children in Haiti in 2014. PLOS Neglected Tropical Diseases 15(6): e0009494. https://doi.org/10.1371/journal.pntd.0009494
	10.3390/v13112293	Saivish MV, Gomes da Costa V, de Lima Menezes G, Alves da Silva R, Dutra da Silva GC, Moreli ML, Sacchetto L, Pacca CC, Vasilakis N, Nogueira ML. Rocio Virus: An Updated View on an Elusive Flavivirus. Viruses. 2021; 13(11):2293
	10.1093/jmedent/1.1.50	Thomas H. G. Aitken, Leslie Spence, Raymond Manuel, Virus Transmission Studies With Trinidadian Mosquitoes Part Iv. Kairi Virus, Journal of Medical Entomology, Volume 1, Issue 1, 1 March 1964, Pages 50–52
	10.3390/v15040843	Ali I, Alarcón-Elbal PM, Mundle M, Noble SAA, Oura CAL, Anzinger JJ, Sandiford SL. The Others: A Systematic Review of the Lesser-Known Arboviruses of the Insular Caribbean. Viruses. 2023; 15(4):843
Ae. serratus	10.1093/jmedent/6.2.207	Aitken THG, Spence L, Jonkers AH, Downs WG. A 10-year survey of Trinidadian arthropods for natural virus infections (1953–1963). J Med Entomol 1969; 6:207–215.
	10.4269/ajtmh.1965.14.460	Sellers RF, Bergold GH, Sua´rez OM, Morales A. Investigations during Venezuelan equine encephalitis outbreaks in Venezuela—1962–1964. Am J Trop Med Hyg 1965; 14:460–469
	10.1590/S0036-46651991000600007	Vasconcelos, P. F. da C., Travassos da Rosa, J. F. S., Travassos da Rosa, A. P. de A., Dégallier, N., Pinheiro, F. de P., & Sá filho, G. C.. (1991). Epidemiologia das encefalites por arbovírus na amazônia brasileira. Revista Do Instituto De Medicina Tropical De São Paulo, 33(6), 465–476
	10.3201/eid1612.100608	Cardoso Jda C, de Almeida MA, dos Santos E, da Fonseca DF, Sallum MA, Noll CA, Monteiro HA, Cruz AC, Carvalho VL, Pinto EV, Castro FC, Nunes Neto JP, Segura MN, Vasconcelos PF. Yellow fever virus in Haemagogus leucocelaenus and Aedes serratus mosquitoes, southern Brazil, 2008. Emerg Infect Dis. 2010 Dec;16(12):1918-24.
	10.2987/12-6222R.1	Marchi MJ, Pereira PA, de Menezes RM, Tubaki RM. New records of mosquitoes carrying Dermatobia hominis eggs in the state of São Paulo, southeastern Brazil. J Am Mosq Control Assoc. 2012 Jun;28(2):116-8
	10.1371/journal.pone.0246932	Pereira-Silva JW, Ríos-Velásquez CM, Lima GRd, Marialva dos Santos EF, Belchior HCM, et al. (2021) Distribution and diversity of mosquitoes and Oropouche-like virus infection rates in an Amazonian rural settlement. PLOS ONE 16(2): e0246932
	https://horizon.documentation.ird.fr/exl-doc/pleins_textes/pleins_textes_6/b_fdi_33-34/38274.pdf	Degallier N, Travassos da Rosa APA, Vasconcelos PFC, Herve JP, Sa Filho GC, Travassos da Rosa ES, Travassos da Rosa JFS, Rodrigues SG. Modifications of arbovirus transmission in relation to construction of dams in Brazilia Amazonia. Public health in the amazon 1992; 44:124-135.
	10.1603/0022-2585(2005)042[0891:IOVFMD]2.0.CO;2	Turell MJ, O'Guinn ML, Jones JW, Sardelis MR, Dohm DJ, Watts DM, Fernandez R, Travassos da Rosa A, Guzman H, Tesh R, Rossi CA, Ludwig V, Mangiafico JA, Kondig J, Wasieloski LP Jr, Pecor J, Zyzak M, Schoeler G, Mores CN, Calampa C, Lee JS, Klein TA. Isolation of viruses from mosquitoes (Diptera: Culicidae) collected in the Amazon Basin region of Peru. J Med Entomol. 2005 Sep;42(5):891-8.
	10.1371/journal.pntd.0009494	Elbadry MA, Durães-Carvalho R, Blohm GM, Stephenson CJ, Loeb JC, et al. (2021) Orthobunyaviruses in the Caribbean: Melao and Oropouche virus infections in school children in Haiti in 2014. PLOS Neglected Tropical Diseases 15(6): e0009494. https://doi.org/10.1371/journal.pntd.0009494
	10.3390/v15040843	Ali I, Alarcón-Elbal PM, Mundle M, Noble SAA, Oura CAL, Anzinger JJ, Sandiford SL. The Others: A Systematic Review of the Lesser-Known Arboviruses of the Insular Caribbean. Viruses. 2023; 15(4):843
	10.3390/v15040843	Ali I, Alarcón-Elbal PM, Mundle M, Noble SAA, Oura CAL, Anzinger JJ, Sandiford SL. The Others: A Systematic Review of the Lesser-Known Arboviruses of the Insular Caribbean. Viruses. 2023; 15(4):843
	10.1093/jmedent/42.5.875	Armstrong PM, Andreadis TG, Anderson JF, Main AJ. Isolations of Potosi virus from mosquitoes (Diptera: Culicidae) collected in Connecticut. J Med Entomol. 2005 Sep;42(5):875-81. doi: 10.1093/jmedent/42.5.875.

Ae. taeniorhynchus	10.1093/jmedent/1.1.50	Aitken THG, Leslie Spence, Raymond Manuel, Virus Transmission Studies With Trinidadian Mosquitoes Part Iv. Kairi Virus, *Journal of Medical Entomology* , Volume 1, Issue 1, 1 March 1964, Pages 50–52
	10.1016/j.virol.2007.10.011	Smith DR, A. Paige Adams, Joan L. Kenney, Eryu Wang, Scott C. Weaver, Venezuelan equine encephalitis virus in the mosquito vector Aedes taeniorhynchus: Infection initiated by a small number of susceptible epithelial cells and a population bottleneck, Virology, Volume 372, Issue 1, 2008,Pages 176-186
	10.1371/journal.pone.0259419	Fish D, Tesh RB, Guzman H, Travassos da Rosa APA, Balta V, et al. (2021) Emergence potential of mosquito-borne arboviruses from the Florida Everglades. PLOS ONE 16(11): e0259419.
	10.1089/vbz.2019.2554	Michael J. Turell, Lee W. Cohnstaedt, and William C. Wilson.Effect of Environmental Temperature on the Ability of Culex tarsalis and Aedes taeniorhynchus (Diptera: Culicidae) to Transmit Rift Valley Fever Virus.Vector-Borne and Zoonotic Diseases.Jun 2020.454-460.
	10.1016/0035-9203(71)90089-7	Bertram DS. Mosquitoes of British Honduras, with some comments on malaria, and on arbovirus antibodies in man and equines. Trans R Soc Trop Med Hyg. 1971;65(6):742-62.
Ae. aegypti	https://pubmed.ncbi.nlm.nih.gov/13207100/	Chamberlain EW, Kissling EE, Sikes EK. Studies on the North American arthropod-borne encephalitides. VII. Estimation of amount of Eastern equine encephalitis virus inoculated by infected Aedes aegypti. Am J Hyg 1954; 60:286–291.
	10.1093/jmedent/6.2.207	Aitken THG, Spence L, Jonkers AH, Downs WG. A 10-year survey of Trinidadian arthropods for natural virus infections (1953–1963). J Med Entomol 1969; 6:207–215.
	10.1093/jmedent/31.1.123	Nasci RS, Mitchell CJ. Larval diet, adult size, and susceptibility of Aedes aegypti (Diptera: Culicidae) to infection with Ross river virus. J Med Entomol 1994; 31:123–126
	10.1590/s0074-02761999000400026	Brito AC, Fontes G, Rocha EM, Rocha DA, Regis L. Development of Dirofilaria immitis (Leidy) in Aedes aegypti (L.) and Culex quinquefasciatus (say) from Maceió, Alagoas, Brazil. Mem Inst Oswaldo Cruz. 1999 Jul-Aug;94(4):575-6
	10.1673/031.007.5501	Lutomiah JJL, Mwandawiro C, Magambo J, Sang RC. Infection and vertical transmission of Kamiti river virus in laboratory bred Aedes aegypti mosquitoes. J Insect Sci 2007; 7:1–7
	10.1093/jmedent/45.6.1117	Ortiz DI, Kang W, Weaver SC. Susceptibility of Ae. aegypti (Diptera: Culicidae) to infection with epidemic (subtype IC) and enzootic (subtypes ID, IIIC, IIID) Venezuelan equine encephalitis complex alphaviruses. J Med Entomol. 2008 Nov;45(6):1117-25
	10.4269/ajtmh.2011.11-0359	Long KC, Ziegler SA, Thangamani S, Hausser NL, Kochel TJ, Higgs S, Tesh RB. Experimental transmission of Mayaro virus by Aedes aegypti. Am J Trop Med Hyg. 2011 Oct;85(4):750-7.
	10.1089/vbz.2011.0660	Wang Z, Zhang X, Li C, Zhang Y, Xing D, Wu Y, Zhao T. Vector competence of five common mosquito species in the People's Republic of China for Western equine encephalitis virus. Vector Borne Zoonotic Dis. 2012 Jul;12(7):605-8.
	10.1128/JVI.00370-14	Vega-Rúa A, Zouache K, Girod R, Failloux AB, Lourenço-de-Oliveira R. High level of vector competence of Aedes aegypti and Aedes albopictus from ten American countries as a crucial factor in the spread of Chikungunya virus. J Virol. 2014 Jun;88(11):6294-306.
	10.1371/journal.pntd.0004543	Chouin-Carneiro T, Vega-Rua A, Vazeille M, Yebakima A, Girod R, et al. (2016) Differential Susceptibilities of *Aedes aegypti* and *Aedes albopictus* from the Americas to Zika Virus. PLOS Neglected Tropical Diseases 10(3): e0004543
	10.1186/s13071-019-3643-0	Ayers, V.B., Huang, YJ.S., Lyons, A.C. *et al.* Infection and transmission of Cache Valley virus by *Aedes albopictus* and *Aedes aegypti* mosquitoes. *Parasites Vectors* **12**, 384 (2019).
	10.1080/22221751.2019.1688097	Kamgang B, Vazeille M, Yougang AP, Tedjou AN, Wilson-Bahun TA, Mousson L, Wondji CS, Failloux AB. Potential of *Aedes albopictus* and *Aedes aegypti* (Diptera: Culicidae) to transmit yellow fever virus in urban areas in Central Africa. Emerg Microbes Infect. 2019;8(1):1636-1641
	10.1146/annurev-ento-011019-024918	Brady OJ, Hay SI. The Global Expansion of Dengue: How *Aedes aegypti* Mosquitoes Enabled the First Pandemic Arbovirus. Annu Rev Entomol. 2020 Jan 7;65:191-208
		CDC: Centers for Disease Control and Prevention. Mosquito species in which West Nile virus has detected, United States, 199–2016 (reported to ArboNET). Available at https://www.cdc.gov/westnile/resources/pdfs/MosquitoSpecies1999-2016.pdf (Accessed December 3, 2020).
Ps. albipes	10.3390/v15040843	Ali I, Alarcón-Elbal PM, Mundle M, Noble SAA, Oura CAL, Anzinger JJ, Sandiford SL. The Others: A Systematic Review of the Lesser-Known Arboviruses of the Insular Caribbean. Viruses. 2023; 15(4):843
	10.1146/annurev.mi.14.100160.001401	Kissling RE. The arthropod-borne viruses of man and other animals. Annu Rev Microbiol 1960; 14:261–282

Ps. albipes	10.4269/ajtmh.1959.8.175	GROOT H, KERR JA, SANMARTIN C, VIDALES H. Antibodies to yellow fever and other arthropod-borne viruses in human residents of San Vicente de Chucuri, Santander, Colombia. Am J Trop Med Hyg. 1959 Mar;8(2 Pt 1):175-89.
Ps. ferox	https://horizon.documentation.ird.fr/exl-doc/pleins_textes/pleins_textes_6/b_fdi_33-34/38274.pdf	Degallier N, Travassos da Rosa APA, Vasconcelos PFC, Herve JP, Sa Filho GC, Travassos da Rosa ES, Travassos da Rosa JFS, Rodrigues SG. Modifications of arbovirus transmission in relation to construction of dams in Brazilia Amazonia. Public health in the amazon 1992; 44:124-135.
	10.1603/0022-2585-37.6.835	M. J. Turell, J. W. Jones, M. R. Sardelis, D. J. Dohm, R. E. Coleman, D. M. Watts, R. Fernandez, C. Calampa, T. A. Klein, Vector Competence of Peruvian Mosquitoes (Diptera: Culicidae) for Epizootic and Enzootic Strains of Venezuelan Equine Encephalomyelitis Virus , *Journal of Medical Entomology* , Volume 37, Issue 6, 1 November 2000, Pages 835–839
	10.1371/journal.pntd.0009494	Elbadry MA, Durães-Carvalho R, Blohm GM, Stephenson CJ, Loeb JC, et al. (2021) Orthobunyaviruses in the Caribbean: Melao and Oropouche virus infections in school children in Haiti in 2014. PLOS Neglected Tropical Diseases 15(6): e0009494. https://doi.org/10.1371/journal.pntd.0009494
	10.3390/v13112293	Saivish MV, Gomes da Costa V, de Lima Menezes G, Alves da Silva R, Dutra da Silva GC, Moreli ML, Sacchetto L, Pacca CC, Vasilakis N, Nogueira ML. Rocio Virus: An Updated View on an Elusive Flavivirus. Viruses. 2021; 13(11):2293
	10.1093/jmedent/42.5.875	Armstrong PM, Andreadis TG, Anderson JF, Main AJ. Isolations of Potosi virus from mosquitoes (Diptera: Culicidae) collected in Connecticut. J Med Entomol. 2005 Sep;42(5):875-81. doi: 10.1093/jmedent/42.5.875.
	https://doi.org/10.3390/v15040843	Ali I, Alarcón-Elbal PM, Mundle M, Noble SAA, Oura CAL, Anzinger JJ, Sandiford SL. The Others: A Systematic Review of the Lesser-Known Arboviruses of the Insular Caribbean. Viruses. 2023; 15(4):843
Ps. ciliata	10.32473/EDIS-IN967-2012	Ragasa, E.V., & Kaufman, P.E. (2013). A Mosquito Psorophora ciliata (Fabricius) (Insecta: Diptera: Culicidae).
	10.1111/mve.12069	Paras KL, O'Brien VA, Reiskind MH. 2014. Comparison of the vector potential of different mosquito species for the transmission of heartworm, *Dirofilaria immitis* , in rural and urban areas in and surrounding Stillwater, Oklahoma, U.S.A. Medical and Veterinary Entomology 28: 60-67. DOI: 10.1111/mve.12069
		CDC: Centers for Disease Control and Prevention. Mosquito species in which West Nile virus has detected, United States, 199–2016 (reported to ArboNET). Available at https://www.cdc.gov/westnile/resources/pdfs/MosquitoSpecies1999-2016.pdf (Accessed December 3, 2020).
Ps. cilipes	10.1016/0035-9203(71)90089-7	Bertram DS. Mosquitoes of British Honduras, with some comments on malaria, and on arbovirus antibodies in man and equines. Trans R Soc Trop Med Hyg. 1971;65(6):742-62.
Cx. coronator	10.3390/v15040843	Ali I, Alarcón-Elbal PM, Mundle M, Noble SAA, Oura CAL, Anzinger JJ, Sandiford SL. The Others: A Systematic Review of the Lesser-Known Arboviruses of the Insular Caribbean. Viruses. 2023; 15(4):843
	10.4269/ajtmh.1971.20.969	Scherer WF, Dickerman RW, Diaz-Najera A, Ward BA, Miller MH, Schaffer PA. Ecologic studies of Venezuelan encephalitis virus in southeastern México. 3. Infection of mosquitoes. Am J Trop Med Hyg. 1971 Nov;20(6):969-79.
	10.1590/S0036-46651991000600007	Vasconcelos, P. F. da C., Travassos da Rosa, J. F. S., Travassos da Rosa, A. P. de A., Dégallier, N., Pinheiro, F. de P., & Sá filho, G. C.. (1991). Epidemiologia das encefalites por arbovírus na amazônia brasileira. Revista Do Instituto De Medicina Tropical De São Paulo, 33(6), 465–476
	10.1038/s41598-017-18682-3	Elizondo-Quiroga D, Medina-Sánchez A, Sánchez-González JM, Eckert KA, Villalobos-Sánchez E, Navarro-Zúñiga AR, Sánchez-Tejeda G, Correa-Morales F, González-Acosta C, Arias CF, López S, Del Ángel RM, Pando-Robles V, Elizondo-Quiroga AE. Zika Virus in Salivary Glands of Five Different Species of Wild-Caught Mosquitoes from Mexico. Sci Rep. 2018 Jan 16;8(1):809. doi: 10.1038/s41598-017-18682-3. Erratum in: Sci Rep. 2018 May 15;8(1):7887
		CDC: Centers for Disease Control and Prevention. Mosquito species in which West Nile virus has detected, United States, 199–2016 (reported to ArboNET). Available at https://www.cdc.gov/westnile/resources/pdfs/MosquitoSpecies1999-2016.pdf (Accessed December 3, 2020).
	10.1007/s00705-010-0665-5	Saiyasombat R, Dorman KS, Garcia-Rejon JE, Loroño-Pino MA, Farfan-Ale JA, Blitvich BJ. Isolation and sequence analysis of Culex flavivirus from Culex interrogator and Culex quinquefasciatus in the Yucatan Peninsula of Mexico. Arch Virol. 2010 Jun;155(6):983-6

Cx. interrogator	10.1089/vbz.2008.0087	Ulloa A, Ferguson HH, Méndez-Sánchez JD, Danis-Lozano R, Casas-Martínez M, Bond JG, García-Zebadúa JC, Orozco-Bonilla A, Juárez-Ordaz JA, Farfan-Ale JA, García-Rejón JE, Rosado-Paredes EP, Edwards E, Komar N, Hassan HK, Unnasch TR, Rodríguez-Pérez MA. West Nile virus activity in mosquitoes and domestic animals in Chiapas, México. Vector Borne Zoonotic Dis. 2009 Oct;9(5):555-60.
Cx. nigripalpus	10.3390/v15040843	Ali I, Alarcón-Elbal PM, Mundle M, Noble SAA, Oura CAL, Anzinger JJ, Sandiford SL. The Others: A Systematic Review of the Lesser-Known Arboviruses of the Insular Caribbean. Viruses. 2023; 15(4):843
	10.1093/oxfordjournals.aje.a121234	W. D. SUDIA, R. D. LORD, V. F. NEWHOUSE, D. L. MILLER, R. E. KISSLING, VECTOR-HOST STUDIES OF AN EPIZOOTIC OF VENEZUELAN EQUINE ENCEPHALOMYELITIS IN GUATEMALA, 1969, *American Journal of Epidemiology* , Volume 93, Issue 2, February 1971, Pages 137–143
	https://pubmed.ncbi.nlm.nih.gov/8827602/	Mitchell CJ, Morris CD, Smith GC, Karabatsos N, Vanlandingham D, Cody E. Arboviruses associated with mosquitoes from nine Florida counties during 1993. J Am Mosq Control Assoc. 1996 Jun;12(2 Pt 1):255-62
		Lok JB, Walker ED, Scoles GA. Filariasis. In: Eldridge BF, Edman JD, eds. Medical Entomology. Dordrecht: Springer, 2000:299–375.
	10.3201/eid0806.010417	Shaman J, Day JF, Stieglitz M. Drought-induced amplification of Saint Louis encephalitis virus, Florida. Emerg Infect Dis. 2002 Jun;8(6):575-80.
	10.4269/ajtmh.2008.78.666	Barrera R, Hunsperger E, Muñoz-Jordán JL, Amador M, Diaz A, Smith J, Bessoff K, Beltran M, Vergne E, Verduin M, Lambert A, Sun W. First isolation of West Nile virus in the Caribbean. Am J Trop Med Hyg. 2008 Apr;78(4):666-8.
	10.4269/ajtmh.2009.81.452	Cohen SB, Lewoczko K, Huddleston DB, Moody E, Mukherjee S, Dunn JR, Jones TF, Wilson R, Moncayo AC. Host feeding patterns of potential vectors of eastern equine encephalitis virus at an epizootic focus in Tennessee. Am J Trop Med Hyg. 2009 Sep;81(3):452-6
	10.1089/vbz.2008.0087	Ulloa A, Ferguson HH, Méndez-Sánchez JD, Danis-Lozano R, Casas-Martínez M, Bond JG, García-Zebadúa JC, Orozco-Bonilla A, Juárez-Ordaz JA, Farfan-Ale JA, García-Rejón JE, Rosado-Paredes EP, Edwards E, Komar N, Hassan HK, Unnasch TR, Rodríguez-Pérez MA. West Nile virus activity in mosquitoes and domestic animals in Chiapas, México. Vector Borne Zoonotic Dis. 2009 Oct;9(5):555-60.
	10.4269/ajtmh.2011.11-0094	Deardorff ER, Estrada-Franco JG, Freier JE, Navarro-Lopez R, Travassos Da Rosa A, Tesh RB, Weaver SC. Candidate vectors and rodent hosts of Venezuelan equine encephalitis virus, Chiapas, 2006-2007. Am J Trop Med Hyg. 2011 Dec;85(6):1146-53
	10.1603/me13049	Turell MJ, Britch SC, Aldridge RL, Kline DL, et al. Potential for mosquitoes (Diptera: Culicidae) from Florida transmit Rift Valley fever virus. J Med Entomol 2013;50:1111-1117.
Cx. taeniopus	10.3390/v15040843	Ali I, Alarcón-Elbal PM, Mundle M, Noble SAA, Oura CAL, Anzinger JJ, Sandiford SL. The Others: A Systematic Review of the Lesser-Known Arboviruses of the Insular Caribbean. Viruses. 2023; 15(4):843
	10.4269/ajtmh.2011.11-0094	Deardorff ER, Estrada-Franco JG, Freier JE, Navarro-Lopez R, Travassos Da Rosa A, Tesh RB, Weaver SC. Candidate vectors and rodent hosts of Venezuelan equine encephalitis virus, Chiapas, 2006-2007. Am J Trop Med Hyg. 2011 Dec;85(6):1146-53
	10.4269/ajtmh.1984.33.953	Weaver SC, Scherer WF, Cupp EW, Castello DA. Barriers to dissemination of Venezuelan encephalitis viruses in the Middle American enzootic vector mosquito, Culex (Melanoconion) taeniopus. Am J Trop Med Hyg. 1984 Sep;33(5):953-60.
	0.4269/ajtmh.2010.09-0024	Deardorff ER, Forrester NL, Travassos da Rosa AP, Estrada-Franco JG, Navarro-Lopez R, Tesh RB, Weaver SC. Experimental infections of Oryzomys couesi with sympatric arboviruses from Mexico. Am J Trop Med Hyg. 2010 Feb;82(2):350-3.
De. pseudes	10.1603/0022-2585-40.3.306	Turell MJ, O'Guinn ML, Navarro R, Romero G, Estrada-Franco JG. Vector competence of Mexican and Honduran mosquitoes (Diptera: Culicidae) for enzootic (IE) and epizootic (IC) strains of Venezuelan equine encephalomyelitis virus. J Med Entomol. 2003 May;40(3):306-10
		Adames, A.J. 1971. Mosquitoes studies (Diptera, Culicidae) XXIV A revision of the crabhole mosquitoes of the genus Deinocerites. Contributions of the American Entomological Institute 7 (2): 1-156
	10.4269/ajtmh.16-0672	Travassos da Rosa JF, de Souza WM, Pinheiro FP, Figueiredo ML, Cardoso JF, Acrani GO, Nunes MRT. Oropouche Virus: Clinical, Epidemiological, and Molecular Aspects of a Neglected Orthobunyavirus. Am J Trop Med Hyg. 2017 May;96(5):1019-1030.
	10.1146/annurev.mi.14.100160.001401	Kissling RE. The arthropod-borne viruses of man and other animals. Annu Rev Microbiol 1960; 14:261–282

Cq. venezuelenzis	http://ve.scielo.org/scielo.php?script=sci_arttext&pid=S1315-01622014000200002	CDC: Centers for Disease Control and Prevention. Mosquito species in which West Nile virus has detected, United States, 199–2016 (reported to ArboNET). Available at https://www.cdc.gov/westnile/resources/pdfs/MosquitoSpecies1999-2016.pdf (Accessed December 3, 2020).<br>Velásquez, G., (2014). Bionomía, ecología e importancia médica de Coquillettidea Rhynchotaenia venezuelensis Theobald, 1912 (Díptera: Culicidae). *SABER. Revista Multidisciplinaria del Consejo de Investigación de la Universidad de Oriente, 26* (2), 105-113.
Ma. dyari	10.1603/me13049	Turell MJ, Britch SC, Aldridge RL, Kline DL, Boohene C, Linthicum KJ. Potential for mosquitoes (Diptera: Culicidae) from Florida to transmit Rift Valley fever virus. J Med Entomol. 2013 Sep;50(5):1111-7.
Li. durhamii	10.3390/v15040843<br>10.2987/12-6222R.1	Ali I, Alarcón-Elbal PM, Mundle M, Noble SAA, Oura CAL, Anzinger JJ, Sandiford SL. The Others: A Systematic Review of the Lesser-Known Arboviruses of the Insular Caribbean. Viruses. 2023; 15(4):843<br>Habits of some mosquito hosts of VEE (Mucambo) virus from northeastern South America, including Trinidad. In: Proceedings of the Workshop-Symposium on Venezuelan Encephalitis Virus. Washington D.C., September 1971. Pan American Health Organization, 1972:254–256.<br>Marchi MJ, Pereira PA, de Menezes RM, Tubaki RM. New records of mosquitoes carrying Dermatobia hominis eggs in the state of São Paulo, southeastern Brazil. J Am Mosq Control Assoc. 2012 Jun;28(2):116-8
