# Supplementary material for: Abundance and diversity of host-seeking adult female mosquitoes in a coastal ecosystem in southern Mexico
Source: PLoS Negl Trop Dis. 2025 Jun 9;19(6):e0012316. doi: 10.1371/journal.pntd.0012316 (PMC12173415; doi:10.1371/journal.pntd.0012316)
Supplement: Attachment — Submitted filename: Canales-Delgadillo_PLoSNTD_review.docx [file pntd.0012316.s005.docx]

Dear authors,

Your manuscript reports an impressive amount of mosquito sampling and identification, which add substantively to the current literature on mosquito diversity on the Yucatan Peninsula.

This paper could be improved by enhancing the quality of the writing to increase clarity. I provide some examples below, and hope that this will help to guide your edits to the paper. Some of the issues require changes to the order in which information is presented, especially in the introduction. Other issues require refining the writing to improve clarity and reduce redundancies. There are also grammatical errors throughout (for example, in the title, it should be “.. in a semi-conserved area **in** southern Mexico”).

Regarding the statistical analysis, I believe that a generalized linear mixed model would be a more appropriate analysis approach than Kruskal-Wallis because it would allow you to account for pseudo-replication by transect site, and would also allow you to include various parameters of interest in the same model.

I believe that your data is important to publish, and I encourage you to make the suggested changes to the writing and data analysis! Please see my more detailed comments below:

22-23: This line is overly general. I suggest making your first sentence more informative about your specific study subject.

31: Provide a brief explanation of what Hill numbers are ; e.g. “We calculated Hill numbers to quantify biodiversity using incidence data…”

Introduction: The introduction section does not flow logically; I suggest reordering (e.g. start with an explanation of vector-borne diseases and zoonoses, then discuss mosquito diversity, and then the impact of the host community, and finally discuss the unique ecology and background info pertaining to the location.) This is just an example of a way that you could reorder it.

Make sure you provide all the necessary info for a reader to fully understand (for example, when you mention aegypti and albimanus, you do not explain that these are well described species because they are known vectors of dengue and malaria.

The writing needs to be tightened up; for example, lines 70-76 is very repetitive and could be edited to just one sentence. Also 85-87 is repetitive with 70-72. These are just a couple of examples, but there are similar types of repetitive writing throughout the paper. There are also typos (e.g. 116-118 should be “area” and “mangrove”).

121: how did you calculate an average distance from a large rectangle with multiple sampling points?

140: What do ECDC and EFSA stand for?

143: Who were the collectors? Authors of the paper, volunteers, or employees? Did collectors collect off of themselves or off of each other? How many went out at a time? Was there a human subjects approval (e.g. IRB in the USA, not sure what the equivalent is in Mexico)?

151: Can you provide more information about how they walked and collected off of themselves at the same time? Was there any control for how long/fast they walked for?

159: Were mosquitoes trapped near dogs held separately to see if there were differences in species attracted to dogs and humans? If not, then it isn’t quite accurate to say you were looking just at anthropophagous mosquitoes, but rather host-seeking mosquitoes.

163-165: How did you use so many keys? Did you use different keys for different genera? Or what was the approach?

170: “seasonal pattern” isn’t the correct term to use here; I suggest using: “… according to the season of each collection event (DS, RS, NS) ….”

179: I think it would be more appropriate to use a generalized linear mixed model so that you can include site as a random effect; you have pseudo-replication of your data points by transect within your study. This would also allow you to include season and hour into the same model.

182-184: I think that the this would probably be better to include as part of your discussion, but if you want to keep it as part of your methods/results, then I suggest separating the mention of the literature review into a new paragraph and keeping your statement about R connected to your description of the stats.

200-202: You did not mention that you also collected from buildings and plants in your methods. How was this done? Please provide more info about this.

Table 1: can you please provide a citation for each mosquito- pathogen association?

Why do you include species richness in the title? I would focus your title on seasonal and temporal abundance by mosquito species.

You wrote “anthropophagous” mosquitoes in the title; does this mean that this does not include any mosquitoes collected on dogs or plants/buildings?

I think it would be helpful to have another column that gives the total by season across all day times.

Would it be potentially easier to read if you divide the hours into groups of day, night, and dawn/dusk? The downside of this approach is that readers would have less detail, but you could include the full table in supplemental.

218-219: it is confusing whether this list is of genera that were or were not present, please clarify.

Figure 2: it is hard to see the 6 hours; again, it might be better to group by day, night and dusk/dawn as appropriate.

This is the first mention of the three vegetation type groups with acronyms; please define.

I don’t understand what the Renyi diversity profile is or how it was created; can you please provide more information?

247: How was the diversity analysis conducted? Also, in general, the R packages/ functions that were used should be cited in your methods.

253-254: Significance and overlapping of confidence intervals are not synonymous in most analyses; if this is different for Hill numbers, please provide a reference

255: Not clear according to what test?

Figure 4: numbers overlap the points, making it hard to read.

Table 3: I still believe that it would be better to discuss comparisons between your study and other previous studies in the discussion, but if you decide to keep this table, please add references for each of the other locations.

Did the other locations have a eudominant species other than *Ae. taeniorhynchus?*

289: I suggest providing more info about the other papers; e.g. what kind of collection method did they use? Which seasons did they sample in?

295: You almost never use this acronym in the paper; I suggest not using it and instead writing it out. I forgot what it was and had to go looking for the definition in the intro.

302: where?

321-323: you may want to remind readers what the temps were for each season here so reader can compare to the ranges you just presented.

329: what do you mean by between? You only sampled at 5 and 9 am, so you don’t have data in between, right?

333: dusk is just during the time when the sun is setting, not the whole night.

Also, can you comment whether these diurnal mosquitoes were also observed during the day? What about other observations of aegypti and other species feeding at night in other publications?

340-342: I don’t understand this statement; how is the presence of collectors any different than the presence of any human?

345: I would assume this is an overestimate of coverage given that the collection method is biased towards mosquitoes that feed on humans, so there are potentially even more mosquito species that weren’t captured because of the HLC bias. I think you should acknowledge this.

363: which species? This is not specific enough to be informative.

371: please state which pathogens here in the discussion ( and cite literature that demonstrates role as vector)

373: It is also a major nuisance mosquito, which can have public health impacts by reducing time spent outdoors.

376: “It is important to…” should be removed here and other places where it is used as an introduction to something you are stating; we can assume anything that you are highlighting is important!

386-387: how does it suggest this? I don’t think that this necessarily provides any indication of whether sites were natural or artificial. Please clarify.

380: can you provide details regarding how far from human residences the *Ae. aegypti* were collected please? This would help to understand whether the mosquitoes were breeding in the forested habitat or potentially flew from nearby houses.

Careful to make sure all mentions of *Ae. aegypti* are written properly with correct capitalization, spelling, and italics.

401: I don’t understand this sentence; do you mean that only one of all the YP studies did not report the presence of *Haemagogus* species? Or did they find more/fewer species? Please clarify. – After looking at the reference, I see that the Carpio-Orantes paper is a review paper; this was unclear in the first reading of this paragraph. I suggest rewording.

412: which two species?

445: In English, mosquito exclusively refers to the Culicidae family; suggest rewording.

450-451: statements such as this one should have a citation.

453: It doesn’t really standout; it seems pretty average based on the data from other locations.

459: Dengue should be lowercase

470: Should be “funding”

473: According to PLoS NTD author guidelines: “All data and related metadata underlying the findings reported in a submitted manuscript should be deposited in an appropriate public repository”.

References: Make sure species names are italicized in references.

Discussion: it would be good to mention somewhere that you only conducted this study for one year and that most seasonal pattern studies should be conducted over at least 2 years. I understand that time/budget may have been prohibitive; this data is definitely still worth publishing, but this limitation should be noted.
