# Supplementary material for: Abundance and diversity of host-seeking adult female mosquitoes in a coastal ecosystem in southern Mexico
Source: PLoS Negl Trop Dis. 2025 Jun 9;19(6):e0012316. doi: 10.1371/journal.pntd.0012316 (PMC12173415; doi:10.1371/journal.pntd.0012316)
Supplement: Attachment — Submitted filename: Response_to_reviewers.docx [file pntd.0012316.s006.docx]

**Dear Editor of Plos Neglected Tropical Diseases,**

We are thankful for the revision made to our manuscript. We carefully revised and attended to each comment and recommendation from you and the reviewers to improve our work and increase the odds of being accepted for publication in the journal PLOS Neglected Tropical Diseases.

Below, you will find a response to each comment and the changes made to the original manuscript.

**Additional Editor Comments:**

*The manuscript has interesting and unique information on Yucatan peninsula mosquito diversity and is potentially acceptable for publication, however, all three reviewers had several comments and suggestions to improve its quality. Two of them recommended a major revision and one a minor one. Overall, reviewers suggested to revise the introduction for clarity, improve methods section by providing more details, address whether IRB approval was obtained for human landing collections, perform GLM instead of non-parametrical tests, improve clarity of results and discussion sections considering that sampling did not only target anthropophagous mosquitoes among other relevant recommendations. Two reviewers expressed editorial concerns that include considerable editing and to re-analyze the data. I agree with reviewer's comments and would like to request a major revision. This revision shall address all the issues raised by reviewers. Regarding data analysis, I recommend performing GLM and transforming the data, but please provide a justification or alternative if not.*

**Response to Additional Editor Comments:**

As the Editor and the reviewers suggested, we revised the Introduction section. We deleted the irrelevant information and focused on the issues of mosquito species' diversity and ecology and the current knowledge gaps on this issue in some areas of the Yucatan Peninsula, which improves the logical flow of this section. We also addressed the figure issues mentioned; we re-made the Fig. 1 using non-copyrighted base maps and reformatted the all the figures to TIF. Format, dimensions, and figures resolution were validated using PACE V2 (<https://pacev2.apexcovantage.com>). We also followed the recommendation to include GLM in the data analysis instead of using the nonparametric Kruskal−Wallis tests. In addition, in the revised version, we also implemented GEE, PERMANOVA and NMDS analyses to reinforce our analyses and conclusions.

Please provide an Author Summary.

**Response:** We added the Author summary as requested **(L45-61)**.

Please upload all main figures as separate Figure files in .tif or .eps format.

**Response:** We edited/re-made figures to have TIF format and at least 300 dpi resolution. All figures were uploaded as separated files in the revised version of the manuscript.

*Some material included in your submission may be copyrighted*.

**Response:** We re-made Figure 1 using noncopyrighted base maps.

*Data Availability*.

**Response:** We stored row data in a public repository for availability:

<https://doi.org/10.5281/zenodo.14633234>

**Response to Reviewer’s comments**

**METHODS**

**Reviewer #1 comment:**

*The manuscript needs to be substantially focused. Authors need to consider that the journal is related to diseases, a topic they discuss only briefly. They may always opt for a different journal and retain the same analysis.*

**Response:**

We appreciate the reviewer’s feedback regarding the focus of our manuscript. In response, we have made substantial revisions to emphasize the relevance of mosquito diversity in the transmission of diseases such as dengue, Zika, and Chikungunya. We have now provided a clearer discussion on the medical importance of the mosquito species found in our study and their potential roles as vectors of these neglected tropical diseases.

Additionally, we have expanded our discussion to highlight the epidemiological significance of our study area **(L429-471)**. The region is characterized by a high rate of immigration (both legal and illegal), which may facilitate the introduction and spread of vector-borne diseases. Furthermore, it serves as a major hotspot for migratory birds from North America, which are known to play a role in the transmission and dispersal of arboviruses such as West Nile virus and equine encephalitis viruses. Given the presence of mosquito species considered competent vectors for these pathogens in our study area, we believe our findings are highly relevant to the readership of PLOS Neglected Tropical Diseases.

We sincerely hope that these substantial modifications demonstrate the alignment of our study with the journal’s scope, emphasizing the public health implications of mosquito diversity and vector-borne disease risks in the region. We appreciate the reviewer’s suggestion and believe that the revised version better reflects the journal’s focus.

*First at all, the Introduction lay out is far from being publishable as it reads quite disorganized and with a lack of flow. The onset talks about topics that are very disparate and not related to what the paper is about. I suggest the authors concentrate on the main aims of the paper (diversity and abundance of mosquitoes) and write their introduction accordingly. For example, the first two paragraphs are really distracting.*

**Response:**

We appreciate the reviewer’s constructive feedback regarding the structure and focus of the Introduction. In response to these concerns, we have thoroughly revised the Introduction to enhance its coherence, logical flow, and direct alignment with the study’s objectives.

Specifically, we have:

1. Streamlined the introduction to focus on the diversity and abundance of mosquitoes, ensuring that all background information is directly relevant to the study.
2. Removed content that was not essential for setting the context of mosquito diversity and vector-borne disease transmission.
3. Reorganized the paragraphs to establish a clearer narrative, beginning with the ecological importance of the study region, followed by the significance of mosquito diversity in disease transmission, and concluding with the rationale for our study.
4. Clarified the knowledge gaps that this study aims to address, ensuring a smooth transition into the research objectives.

**Modified text (L63-97):**

The southern Gulf of Mexico is renowned for its ecological diversity. It encompasses wetlands, jungles, coastal areas, mangroves, and estuaries, providing critical habitats for native and migratory wildlife, including insects [1–3]. Mosquitoes are among the most significant insect vectors of zoonotic diseases, transmitting pathogens affecting human and wildlife health [4–10]. Globally, 3,570 mosquito species have been identified, with approximately 6% occurring in Mexico [11]. These insects are pivotal in transmitting diseases such as malaria, dengue fever, Chikungunya, and Zika virus, which collectively impact millions of people annually, particularly in tropical regions [12–14]. Understanding mosquito diversity and abundance in these areas is essential for developing effective vector-borne disease control strategies [15].

Southern Mexico, including the Yucatan Peninsula, is a hotspot for mosquito-borne diseases due to its tropical climate, high humidity, and abundant water bodies, all of which create ideal conditions for mosquito breeding [16,17]. While some mosquito species, such as *Aedes aegypti* and *Anopheles albimanus*, are well-studied vectors in this region, many other mosquito species’ diversity and ecological roles remain poorly understood [18]. Filling these knowledge gaps is critical for enhancing our understanding of mosquito ecology and improving disease prevention efforts in this tropical region.

Isla del Carmen, located off the coast of Campeche, is characterized by its mangrove ecosystems and humid tropical climate, providing numerous microhabitats for mosquito populations [19–21]. Despite its ecological significance, there is limited information about the diversity of mosquito species on the island. Most studies have focused on mainland Campeche, examining mosquito species in low semi-evergreen forests, mangroves, and agricultural landscapes [22,23]. The only known comprehensive survey of mosquito species on Isla del Carmen was conducted between 1996 and 2006, reporting 14 medically relevant species for urban areas [24]. Land use plays a crucial role in shaping mosquito distribution. Thus, the transformation of natural ecosystems can increase the availability of breeding sites and alter species interactions, as habitat degradation and urbanization often create conditions that favor vector species [25].

To address these gaps, we aimed to determine the diversity of host-seeking female mosquito species on Isla del Carmen. We hypothesized that mosquito species richness and diversity are influenced by seasonality, time of day, and vegetation type, which in turn shape the dynamics of disease risk in the region. Our study provides baseline data to inform future research on mosquito populations and the dynamics of vector-borne diseases under changing climatic and ecological conditions. This knowledge is crucial for designing effective disease control and prevention strategies tailored to the unique environmental characteristics of coastal areas in southern Mexico.

We believe that these modifications have significantly improved the clarity and relevance of the Introduction.

*I have some issues in relation to whether this study fits into de journal scope. To remedy this, authors should emphasize and/or frame their study aims within a zoonotic background, for example mentioning that they investigated mosquitoes of medical importance to inform local health authorities. To be coherent with this, I suggest that authors divide mosquitoes of medical importance vs those that are not in the analysis. This would imply setting new objectives.*

**Response:**

We appreciate the reviewer’s concern regarding the alignment of our study with the journal’s scope and their valuable suggestions to emphasize the zoonotic relevance of our research. In response, we have revised the manuscript to explicitly highlight the medical and veterinary importance of the mosquito species investigated. Specifically, we have:

- Adjusted the study aim to state that we assessed the **diversity, medical, and veterinary relevance** of prey-seeking female mosquito species.
- Emphasized how our findings contribute to understanding the **dynamics of vector-borne diseases** in a changing environment, reinforcing the public health implications of our research.
- Clarified that this study provides **baseline data to inform health authorities and guide future disease prevention strategies** in southern Mexico.

**Modified text (L90-97):**

To address these gaps, we aimed to determine the diversity of host-seeking female mosquito species on Isla del Carmen. We hypothesized that mosquito species richness and diversity are influenced by seasonality, time of day, and vegetation type, which in turn shape the dynamics of disease risk in the region. Our study provides baseline data to inform future research on mosquito populations and the dynamics of vector-borne diseases under changing climatic and ecological conditions. This knowledge is crucial for designing effective disease control and prevention strategies tailored to the unique environmental characteristics of coastal areas in southern Mexico.

*I am not at all happy with the use of non-parametrical tests. Did you try to transform your variables?*

**Response:**

We appreciate the reviewer’s comment regarding the use of non-parametric tests and the suggestion to explore variable transformations. We would like to provide a justification for our analytical approach, including the use of the Kruskal-Wallis test (although removed from the new manuscript’s version), and the subsequent implementation (for the new manuscript’s version) of non-binomial generalized linear models (nb-GLM), generalized estimating equations (GEE) models, Permanova and NMDS analysis.

The Kruskal-Wallis test was initially chosen because our data on mosquito abundance were non-normally distributed, as confirmed by Shapiro-Wilk tests. Non-parametric tests like Kruskal-Wallis are robust to violations of normality and do not require assumptions about the underlying distribution of the data, making them suitable for our dataset. In addition, the data included ordinal and non-linear relationships between variables (e.g., mosquito abundance across seasons and hours), which further supported the use of non-parametric methods.

To address the reviewer’s concern, the potential limitations of the Kruskal-Wallis test (e.g., its inability to account for correlations within the data or handle multiple predictors simultaneously), and further strengthen our analysis, we removed the results from these analyses and implemented a negative-binomial generalized linear models and a generalized estimating equations (GEE) approach.

GEE is particularly well-suited for our study because it accounts for repeated measures and potential correlations within the data (e.g., mosquito captures (abundance) at different hours within the same day or across seasons). Also, it allows us to model the abundance of mosquitoes as a function of multiple predictors (season, vegetation type, and time of day) simultaneously, providing a more comprehensive understanding of the factors influencing mosquito abundance.

GEE is flexible in handling non-normal distributions and can accommodate count data (e.g., mosquito captures) by using link functions such as log-link for Poisson distribution. Unlike traditional parametric tests, GEE does not require strict assumptions about the distribution of the dependent variable, making it a robust choice for our dataset.

Why was variable transformation not our preferred approach? While variable transformation could have been an alternative to address non-normality, it may not have been appropriate for our data. It is known that transformations (e.g., log or square root) can distort the interpretability of the results, especially when dealing with count data with many zero values (common in ecological surveys like ours).

We hope this clarifies our analytical approach and demonstrates the careful consideration given to the reviewer’s feedback.

We are confident that the negative-binomial GLM, the GEE, Permanova and NMDS analyses used in this revised version, strengthen the validity and reliability of our findings.

In correspondence with this, we edited the Analysis data text in the Methods section as follows:

**Modified text (L167-210):**

For diversity analyses, the data were grouped according to season (dry, rainy, and norte) of each collection event and the 24-hour sampling cycle. Species richness was estimated using the function specnumber of the R package vegan [37].

To evaluate the sampling coverage during different seasons and at different times of the 24-hour cycle, we generated accumulation diversity curves. After the conversion of the abundance data to incidence data, we generated three estimates of Hill numbers of order q: 1) species richness (q = 0); 2) Shannon diversity, as the exponential of Shannon entropy or common species (q = 1); and 3) Simpson diversity, calculated as the inverse of the Simpson concentration or dominant species (q = 2), using the functions iNEXT and estimateD in the R package iNEXT, [38,39]. To visualize the diversity patterns of the mosquito community, we calculated the Rényi diversity profiles (α), which provide a comprehensive view of species diversity by incorporating multiple diversity indices into a single framework through the function renyicomp of the BiodivesityR package [40]. When α = 0, the Rényi diversity corresponds to species richness; when α = 1, it approximates the Shannon index; and when α = 2, it is equivalent to the Simpson index, which emphasizes species dominance. As α increases, the diversity becomes more influenced by the most abundant species, allowing for a comparison of evenness across communities. We generated Rényi diversity profiles for each sampling season, hour, and vegetation type to compare mosquito assemblages. The profiles were interpreted by assessing how diversity changed across different α values, with curves that remained above others indicating consistently higher diversity across all scales.

To further analyze the effects of season (norte, dry, and rainy) and sampling hour (01:00, 05:00, 09:00, 13:00, 17:00, and 21:00) on the abundance of host-seeking female mosquitoes, we used a Negative Binomial Generalized Linear Model (GLM) implemented with the glm.nb function from the MASS package [41] in R. This approach was chosen due to the presence of overdispersion in the count data, which violates the assumptions of a Poisson GLM. The Negative Binomial GLM accounts for overdispersion by introducing a dispersion parameter, making it suitable for modeling mosquito abundance data [42]. We applied a Non-Metric Multidimensional Scaling (NMDS) analysis to test clustering patterns across seasons and time of day. NMDS was used to visually explore and reinforce the results of the Negative Binomial GLM, providing a complementary perspective on mosquito activity trends. This ordination approach allows identification of seasonal and diel peaks of organisms’ activity [43], particularly important for areas where species involved in transmitting diseases such as dengue, Zika, and Chikungunya are present. The NMDS was based on a Bray−Curtis dissimilarity matrix derived from species abundance data. The significance of the clustering patterns was tested through a PERMANOVA test using the function adonis2 from the R package vegan [37]. Additionally, to assess the effects of season, time of day, and vegetation type on the abundance of the five most dominant mosquito genera in the study area, we employed a Generalized Estimating Equations (GEE) approach using the R package geepack [44] to analyze phenological patterns. The GEE model was selected because it accounts for correlated data (e.g., repeated measures across time and space) and allows for robust inference in the presence of non-independence [45,46]. Unlike traditional parametric tests, GEE does not require strict assumptions about the distribution of the dependent variable, making it a suitable choice for our dataset. Only genera with more than 60 records over the study period were included in the analysis to ensure reliable estimates. All analyses were performed in R version 4.1.1 [47].

*I suggest adding a specific analysis to detect phenological patterns. This information is key to inform health authorities as for when they should be worried in regards to mosquitoes of medical importance. I am adding a in-revision manuscript from my group that may be of use to authors. Please do not disseminate it as it is not published yet.*

**Response:**

We appreciate the reviewer’s valuable suggestion regarding the importance of detecting phenological patterns in medically relevant mosquito species. To address this issue, in addition to the negative binomial GLM, we have conducted a Non-Metric Multidimensional Scaling (NMDS) analysis in the new manuscript version, which helped analyze the seasonal and hourly patterns of female mosquito activity since NMDS helps identify ecological gradients, making it particularly useful for exploring phenological trends in mosquito populations. The NMDS allowed us to visualize seasonal and diel variations in mosquito activity more clearly. The NMDS also reinforced the findings of the GEE model, which accounts for temporal autocorrelation and statistically evaluates the influence of seasonality and time of day on mosquito occurrence. We believe that, combining these two approaches strengthens our analysis, providing meaningful insights for public health authorities regarding peak periods of mosquito activity and potential disease transmission risks.

We would also like to acknowledge the reviewer’s kind intention to share an in-revision manuscript that may be relevant to our study. However, we were unable to access the document, as we did not find it attached to the review comments or linked within the provided materials. Nonetheless, we truly appreciate the recommendation and remain open to considering its insights if access becomes available.

**Modified text (L187-209):**

To further analyze the effects of season (norte, dry, and rainy) and sampling hour (01:00, 05:00, 09:00, 13:00, 17:00, and 21:00) on the abundance of host-seeking female mosquitoes, we used a Negative Binomial Generalized Linear Model (GLM) implemented with the glm.nb function from the MASS package [41] in R. This approach was chosen due to the presence of overdispersion in the count data, which violates the assumptions of a Poisson GLM. The Negative Binomial GLM accounts for overdispersion by introducing a dispersion parameter, making it suitable for modeling mosquito abundance data [42]. We applied a Non-Metric Multidimensional Scaling (NMDS) analysis to test clustering patterns across seasons and time of day. NMDS was used to visually explore and reinforce the results of the Negative Binomial GLM, providing a complementary perspective on mosquito activity trends. This ordination approach allows identification of seasonal and diel peaks of organisms’ activity [43], particularly important for areas where species involved in transmitting diseases such as dengue, Zika, and Chikungunya are present. The NMDS was based on a Bray−Curtis dissimilarity matrix derived from species abundance data. The significance of the clustering patterns was tested through a PERMANOVA test using the function adonis2 from the R package vegan [37]. Additionally, to assess the effects of season, time of day, and vegetation type on the abundance of the five most dominant mosquito genera in the study area, we employed a Generalized Estimating Equations (GEE) approach using the R package geepack [44] to analyze phenological patterns. The GEE model was selected because it accounts for correlated data (e.g., repeated measures across time and space) and allows for robust inference in the presence of non-independence [45,46]. Unlike traditional parametric tests, GEE does not require strict assumptions about the distribution of the dependent variable, making it a suitable choice for our dataset. Only genera with more than 60 records over the study period were included in the analysis to ensure reliable estimates.

**Reviewer # 2 comments:**

*This manuscript describes a sampling study conducted over a bit more than 1 year in a single area. The study uses the HLCs from 3 individual collectors to calculate a variety of diversity indices. Notably, the biggest issue is the use of the word "anthropophagous" in the title of the study. The methods and results indicate that while HLCs were the predominant collection method, collections were also performed from dogs and inanimate objects which are not in alignment with anthropophagous. This needs to be addressed in revisions. There are also calculated results described that are not described in the methods. Specific comments are:*

*L2: Suggest title change to: "Diversity patterns of female mosquitoes in Southern Mexico." The collections were not performed just of anthrophilic mosquitoes.*

**Response:**

We appreciate the reviewer’s suggestion and acknowledge that our data included mosquito species beyond those with strictly anthropophilic feeding behaviors. To better reflect the scope of our study and highlight the medical and veterinary relevance of the species collected, we have revised the title to **(L1-2)**:

“**Abundance and diversity of host-seeking adult female mosquitoes in a coastal ecosystem in southern Mexico**.”

This revised title more accurately conveys both the ecological and public health implications of our findings.

*L23-24: This sentence could be removed to increase clarity.*

**Response:**

As suggested, we deleted the sentence for clarity.

*L32-33: This sentence is not needed in the Abstract. It is something that is fine to discuss but it is not something that represents a scientific result of this study.*

**Response:**

We appreciate the reviewer’s feedback and agree that the sentence “A literature review determined the diseases associated with the species found.” does not present a direct scientific result of our study. Therefore, we have removed it from the Abstract.

*L37-39: Suggest revision of the conclusion to be more impactful.*

*Text generally: Paragraph formatting is reversed in this review copy from normal paragraph formatting.*

**Response:**

As suggested, we revised the conclusion in the abstract and rewrite it. The new version emphasizes the importance of the findings and connects them to potential applications in disease monitoring and management.

**Modified text (L35-40):**

Isla del Carmen is a key region for mosquito diversity in the Yucatan Peninsula, hosting species known to transmit pathogens to humans and wildlife. Our findings highlight the norte season as a critical period for mosquito activity, emphasizing the need for targeted vector surveillance and control efforts during this time in the region. This study provides valuable insights into mosquito community dynamics and their implications for public health in southern Mexico.

*L56: Format reference to proper callout. Check throughout manuscript.*

**Response:**

We appreciate the reviewer’s observations. We checked out the entire manuscript to fix this mistake.

**Modified text (L67-68):**

Globally, 3,570 mosquito species have been identified, with approximately 6% occurring in Mexico [11].

*Study area: Did the three transects differ in vegetation or were they essentially the same?*

**Response:**

The study area is a 10-ha patch where two main vegetation types coexist (semideciduous forest and mangrove). In addition, an area of buildings with modified and managed vegetation exists there. Sampling transects were established in one of the three vegetation mixtures (accounting for dominant vegetation): buildings and semideciduous forest (BLDSM), mangrove and semideciduous forest (MGSM), semideciduous forest and mangrove (SMMG). This information was provided in lines 130-135:

**Modified text (L130-135):**

The sampling included three collectors that used pooters (oral aspirators) to trap live-flying adult female mosquitoes on three sampling transects established in 1) a vegetation mixture of dominant mangrove and semideciduous forest (MGSM), 2) a mixture of dominant semideciduous and mangrove forest (SMMG), or 3) a semideciduous forest modified by buildings (BLDSM, Fig. 1).

*L116 Should this be Study site and sampling transects?*

**Response:**

We appreciate this observation. Yes, the reviewer is right. We edited the text to fix this mistake.

**Modified text (L106-111):**

**Figure 1. Study site and sampling transects.** The transects were separated by dominant vegetation: semideciduous forest modified by buildings (BLDSM), mangrove and semideciduous forest (MGSM), and semideciduous and mangrove forest (SMMG). The map was created using Microimages software (TNT Mips ver. 2013, Nebraska USA) by masking the study site and sampling transects (https://doi.org/10.5281/zenodo.14633234) on Copernicus Sentinel data 2020 used as a base layer (<https://sentinel.esa.int/documents/247904/690755/Sentinel_Data_Legal_Notice>).

*L121: Suggest revision. Were the human settlements 546m from the perimeter of the sampling area?*

**Response:**

No, the nearest human settlements fall within a radius of about 550m from the center of the sampling area and about 490m from the perimeter of the sampling area. The surrounding landscape of the sampling area includes native non-modified vegetation and disturbed vegetation patches, living areas, university areas, and building development (see image below). The red rectangle is the sampling area. The yellow circle shows the influence area when accounting for a radius of 550m).

*Sampling section: Please specify which transect abbreviation used in figure 1 represents the mangrove, semideciduous, and modified semideciduous. Note the spelling of deciduous.*

**Response:**

We appreciate the reviewer’s comments. In the new manuscript version, the vegetation types and their abbreviations are specified. We also fixed the spelling of semideciduous.

**Modified text (L125-130):**

The sampling included three collectors that used pooters (oral aspirators) to trap live-flying adult female mosquitoes on three sampling transects established in 1) a vegetation mixture of dominant mangrove and semideciduous forest (MGSM), 2) a mixture of dominant semideciduous and mangrove forest (SMMG), or 3) a semideciduous forest modified by buildings (BLDSM, Fig. 1).

*Also, is consent a requirement for HLC studies in Mexico? If so, it should be mentioned.*

**Response:**

Thank you for your thoughtful comment and for raising an important question regarding consent requirements for HLC studies in Mexico. We appreciate the opportunity to clarify this matter.

As you correctly pointed out, consent is a critical ethical consideration in research involving human participants. However, it is important to note that HLC (human landing catch) studies are not explicitly addressed by the Regulations of the General Health Law on Health Research in Mexico. This means that the specific requirements for consent in such studies are not formally outlined within this regulatory framework. Nevertheless, we fully recognize the importance of ethical oversight and transparency in research. In response to your suggestion, we have added text to the manuscript to explicitly clarify this issue and ensure readers are aware of the regulatory context **(L130-141)**. Additionally, we would like to address the ethical oversight of our study. At the time of sampling, our institution did not have an Institutional Review Board (IRB) in place to provide formal approval for research protocols. However, in alignment with our commitment to ethical research practices, we sought and obtained IRB approval *post facto* once the necessary infrastructure was established. This approval confirms that our study, including its methodology and ethical considerations, aligns with internationally recognized standards for research involving human participants. As suggested, we added text to clarify this issue.

**Modified text (L135-146):**

Individuals’ collection was performed mainly through the human landing collection (HLC) method [28]. The HLC technique is one of the oldest methods to catch mosquitoes and has been widely used for its simplicity in collecting host-seeking adult females [28,29]. However, some adult mosquitoes were collected from non-human organisms, including dogs, buildings’ windows and walls, and plant branches and leaves.

According to the Regulations of the General Health Law on Health Research [30], consent for HLC is not required in Mexico. However, all collectors were fully informed about the potential infection risks, and all necessary protective measures were implemented to ensure their well-being and safety during the collection process (use of denim clothes and latex gloves to protect as much skin as possible, IRB approval CEID-SOL-2025). Once the collectors agreed with the working conditions, the sampling began. During the study period, the collectors did not show any infection symptoms.

*L159: If sampling was also sporadically conducted from dogs, then it is not quite accurate to characterize the effort as anthropophagic sampling.*

**Response:**

The reviewer is right. We changed from anthropophagous mosquitoes to host-seeking mosquitoes. This change was applied throughout the text.

*L163-164: Carpenter & LaCasse reference callout?*

**Response:**

We thank the reviewer for this observation. We fixed the correct format of citation.

**Modified text (L160-162):**

for taxonomical identification using specialized keys such as Carpenter and LaCasse [32], Díaz-Nájera [33]…

*L200-201: These collection methods are not mentioned in the sampling section above but should be. These also argue against characterization of the sampling as anthropophagic.*

**Response:**

We agree with the reviewer’s comment. We added the missing information to the sampling section.

**Modified text (L136-139):**

The HLC technique is one of the oldest methods to catch mosquitoes and has been widely used for its simplicity in collecting host-seeking adult females [28,29]. However, some adult mosquitoes were collected from non-human organisms, including dogs, buildings’ windows and walls, and plant branches and leaves.

*Figure 2 (Line 242): Calculation of Renyi diversity profile is not described in the methods. Also, please check spelling in the figure as well as reformat the panels so that they are the same size.*

**Response:**

As suggested, we added the description of Rényi diversity profiles to methods sections. We also remade Figure 2 to have all the panels the same size as pointed out. Also, we checked the spelling and fixed the mistakes. You will find the new figure below this line:

**Modified text (L175-186):**

To visualize the diversity patterns of the mosquito community, we calculated the Rényi diversity profiles (α), which provide a comprehensive view of species diversity by incorporating multiple diversity indices into a single framework through the function *renyicomp* of the BiodivesityR package [40]. When α = 0, the Rényi diversity corresponds to species richness; when α = 1, it approximates the Shannon index; and when α = 2, it is equivalent to the Simpson index, which emphasizes species dominance. As α increases, the diversity becomes more influenced by the most abundant species, allowing for a comparison of evenness across communities. We generated Rényi diversity profiles for each sampling season, hour, and vegetation type to compare mosquito assemblages. The profiles were interpreted by assessing how diversity changed across different α values, with curves that remained above others indicating consistently higher diversity across all scales.

**Reviewer #3 comments:**

*Your manuscript reports an impressive amount of mosquito sampling and identification, which add substantively to the current literature on mosquito diversity on the Yucatan Peninsula. The main weakness is the clarity of the writing, which can be improved through a combination of simple editing and more involved reordering of information (especially in the introduction). The secondary weakness is the statistical analysis, which can be improved through using another analysis method that accounts for pseudo replication (e.g. GLMM) and providing more details about how the current calculations were conducted (e.g. R packages). I believe that your data is important to publish, and I encourage you to make the suggested changes to the writing and data analysis!*

**Response:**

We sincerely appreciate the reviewer’s constructive feedback on the structure and focus of the Introduction. In response to this valuable comment, we have thoroughly revised the Introduction to improve its coherence, logical flow, and alignment with the study’s objectives. To achieve this, we streamlined the content to focus on the diversity and abundance of mosquitoes, ensuring all background information is directly relevant to the study. We removed non-essential details unrelated to mosquito diversity and vector-borne disease transmission, reorganized the paragraphs to establish a clearer narrative, and clarified the knowledge gaps this study aims to address. The revised Introduction **(L62-97)** now begins with the ecological importance of the study region, transitions into the significance of mosquito diversity in disease transmission, and concludes with the rationale for our research, providing a smoother and more focused progression toward the study’s objectives.

We believe that these changes (**see L167-210)** in the new manuscript’s version, and Table 3 below) ensure that our statistical approach is robust, well-documented, and aligned with the study’s objectives.

*The site was clearly described*.

**Response:**

We appreciate that comment.

*Regarding the statistical analysis, I believe that a generalized linear mixed model would be a more appropriate analysis approach than Kruskal-Wallis because it would allow you to account for pseudo-replication by transect site and collection date, and would also allow you to include various parameters of interest in the same model.*

**Response:**

Thank you for your insightful feedback regarding the statistical analysis in our study. We appreciate your suggestion to consider alternative methods, such as Generalized Linear Mixed Models (GLMM), to account for potential pseudoreplication and to provide more details about the analytical procedures. In response to your comment, we have:

- Clarified the rationale for using a Negative Binomial GLM to address overdispersion in mosquito abundance data.
- Provided detailed descriptions of the R packages and functions used for diversity analyses (vegan,iNEXT,BiodiversityR), including Hill numbers and Rényi diversity profiles.
- Explained the use of Non-Metric Multidimensional Scaling (NMDS) and PERMANOVA to explore and validate clustering patterns across seasons and times of day.
- Justified the selection of Generalized Estimating Equations (GEE) to analyze phenological patterns in dominant mosquito genera, emphasizing its suitability for handling correlated data and non-independence.
- Added further details about the statistical methods and R packages to enhance transparency and reproducibility.

All these changes are fully described in the **Methods section (L167-210)**.

*I do not see any mention of an IRB or equivalent review, but human landing catches were utilized to collect mosquitoes, which I believe is considered to involve human subjects.*

**Response:**

We appreciate the reviewer’s comment and fully recognize the importance of ethical oversight and transparency in research. In response to your comment, we have added text to the manuscript to explicitly clarify this issue and ensure readers are aware of the regulatory context **(L140-146)**. Additionally, we would like to address the ethical oversight of our study. At the time of sampling, our institution did not have an Institutional Review Board (IRB) in place to provide formal approval for research protocols. However, in alignment with our commitment to ethical research practices, we sought and obtained IRB approval *post facto* once the necessary infrastructure was established.

**RESULTS**

**Reviewer #1**

*Partly. I'd suggest that the analysis is divided for mosqutioes of medical importance vs mosquitoes of non-medical importance. I also suggest carrying out an analysis of phenology.*

**Response:**

We appreciate the reviewer’s comment. However, we believe that at this stage, dividing the analysis into mosquitoes of medical importance vs. those that are not would require setting new objectives beyond the scope of our current study. Nonetheless, we acknowledge the value of this approach and will consider it in future research efforts. We believe that our revisions sufficiently frame the study within a zoonotic and public health context, ensuring its relevance to the journal’s readership. We also appreciate the reviewer’s valuable suggestion regarding the importance of detecting phenological patterns in medically relevant mosquito species. To address this issue, in addition to the negative binomial GLM, we have conducted a Non-Metric Multidimensional Scaling (NMDS) analysis in the new manuscript’s version, which helped analyze the seasonal and hourly patterns of female mosquito activity since NMDS helps identify ecological gradients, making it particularly useful for exploring phenological trends in mosquito populations.

Full description of the approaches used to analyze phenology are in lines **L187-210**.

**Reviewer #2**

*The results present some issues that must be addressed. See specific comments below.*

*L247: Where are calculations that support this sentence and the others of this paragraph? I did not see them in a Table.*

**Response:**

We appreciate the reviewer’s comment. In the new manuscript’s version, we added, as supplementary material, Table S1 which shows the details of the sample coverage estimations. In addition, we pointed out in the text that Table S1 is available as supplementary material.

**Modified text (L273-278):**

The diversity analysis revealed values higher than 90% sample coverage in the norte and rainy seasons (94.71 and 92.1%, respectively). A lower sample coverage was estimated for the dry season (81.48%) (see supplementary Table S1). In addition, we had sample coverage higher than 80% during each sampling hour (except at 17:00, where the sample coverage was 75.5%), indicating that our sampling effort was sufficient to determine the host-seeking adult female mosquito species richness in our study area.

**Table S1.** Estimations of sample coverage from host-seeking female mosquito incidence data.

|  | T | U | S.obs | SC | Q1 | Q2 | Q3 | Q4 | Q5 | Q6 | Q7 | Q8 | Q9 | Q10 |
| --- | --- | --- | --- | --- | --- | --- | --- | --- | --- | --- | --- | --- | --- | --- |
| **Sampling season** |  |  |  |  |  |  |  |  |  |  |  |  |  |  |
| Norte | 6 | 100 | 28 | 0.947 | 6 | 2 | 5 | 6 | 3 | 6 | 0 | 0 | 0 | 0 |
| Dry | 6 | 18 | 8 | 0.815 | 4 | 2 | 0 | 1 | 0 | 1 | 0 | 0 | 0 | 0 |
| Rainy | 6 | 67 | 20 | 0.921 | 6 | 2 | 4 | 0 | 3 | 5 | 0 | 0 | 0 | 0 |
| **Sampling hour** |  |  |  |  |  |  |  |  |  |  |  |  |  |  |
| 01 | 3 | 36 | 21 | 0.875 | 9 | 9 | 3 | 0 | 0 | 0 | 0 | 0 | 0 | 0 |
| 05 | 3 | 38 | 23 | 0.841 | 11 | 9 | 3 | 0 | 0 | 0 | 0 | 0 | 0 | 0 |
| 09 | 3 | 21 | 13 | 0.857 | 6 | 6 | 1 | 0 | 0 | 0 | 0 | 0 | 0 | 0 |
| 13 | 3 | 22 | 13 | 0.905 | 5 | 7 | 1 | 0 | 0 | 0 | 0 | 0 | 0 | 0 |
| 17 | 3 | 29 | 19 | 0.755 | 11 | 6 | 2 | 0 | 0 | 0 | 0 | 0 | 0 | 0 |
| 21 | 3 | 39 | 25 | 0.812 | 13 | 10 | 2 | 0 | 0 | 0 | 0 | 0 | 0 | 0 |

T = Sample size; U = Total number of incidences; S.obs = Observed species richness; SC = Estimated sample coverage; Q1-Q10 = the first ten counts of incidence frequency.

*L252 & others: Generally, this manuscript would benefit from consistency in the naming between the text and figures. Here, they are NS, RS, DS but on the figure, they are norte, rainy, and dry.*

**Response:**

We thank the reviewer for the comment. We changed the abbreviation NS, RS, and DS for norte, rainy and dry season to be consistent throughout the manuscript.

*L262:* *The text states, "the Hill numbers differed significantly" and then calls to Figure 4A. However, Fig 4A shows diversity, Shannon and simpson with 95% CIs. In the q=0 panel of 4A, the 95% CIs overlap so they are not significantly different. How can you thus conclude they are different? Similarly, most of the 95%CIs overlap in the panels of 4B.*

**Response:**

We appreciate the reviewer’s insightful comment regarding the interpretation of significance in Figure 4A and 4B. After carefully reviewing our data and analyses, we identified the issue and have revised the text accordingly to ensure accuracy. In the original version, we stated that species richness differed significantly; however, as the reviewer correctly pointed out, the 95% confidence intervals (CIs) in the q = 0 panel of Figure 4A overlapped, indicating that the difference was not statistically significant between all seasons. Similarly, we acknowledged that most of the 95% CIs overlapped in Figure 4B, meaning there was no strong statistical support for differences across the 24-hour cycle.

To address this, we have revised the text to accurately reflect our findings and accordingly, we modified the text and improved the figure.

**Modified text (L291-299):**

Rarefaction analysis based on Hill numbers revealed that the species richness differed significantly only between the norte and dry seasons (Figure 4A), indicating a seasonal variation gradient in mosquito community dynamics on Isla del Carmen. Nevertheless*, Ae. taeniorhynchus*, the dominant species, remained highly abundant throughout all seasons, suggesting that annual fluctuations in environmental conditions likely influence the distribution and abundance of the remaining female mosquito species. Although the species richness of host-seeking female mosquitoes appeared to be generally higher between 17:00 and 05:00 hours, no significant differences in species richness, diversity, or dominance were detected across the 24-hour cycle (Figure 4B).

**Improved Figure4:**

*L286: Definitely need to reference those previous studies here. Also needs explanation in the methods section if you are generating these diversity indices from previously published data.*

**Response:** We appreciate the reviewer’s suggestion. In addition to the proper citations in methods section, we added the references of earlier works to the results section in text and in the former Table 3, which become Table 4 in the new manuscript’s version..

**Modified text:**

**Methods (L211-214):** To better comprehend the contribution of the Isla del Carmen mosquito species to regional diversity, we compared our data with diversity reports in other areas of the Yucatan Peninsula. We compiled the published works with available abundance data [22,23,47–53], to estimate species diversity and richness in each state/location as described above.

**Results (L304-309):** According to the reviewed literature [22,23,48–50,52–54], Isla del Carmen is among the places with the greatest species richness on the Yucatan Peninsula, together with Celestún and Calakmul. However, it showed a diversity value similar to that of the urban areas of Merida city [54]. When the eudominant Ae. taeniorhynchus was excluded from the dataset, the diversity of the mosquito community in Isla del Carmen was higher than the estimated value for all other locations (Table 4).

**L310-317:** **Table 4. Comparison of female mosquito species richness (*S_r_*) and diversity of Isla del Carmen and other localities in the Yucatan Peninsula.** Parameters were estimated from the data in this study and in the earlier published works [23,47−49,54]. The estimated Shannon−Wiener diversity index (*H’*) by locality included (*H’_1_*) and excluded (*H’_2_*) *Ae. taeniorhynchus* data to show the effect of this eudominant species on the community ecological signal*.* Capture strategy/months are indicated below table with superscripts.

| Parameter | Isla del Carmen^1^ | Candelaria^2^ |  | Celestún^3^ | Mérida^4^ | Palizada^5^ | Campeche^6^ | Calakmul^7^ | Sian kaan^8^ |
| --- | --- | --- | --- | --- | --- | --- | --- | --- | --- |
| *S_r_* | 26 | 23 |  | 33 | 19 | 11 | 6 | 30 | 26 |
| *H’_1_* | 0.447 | 1.607 |  | 1.629 | 0.751 | 1.377 | 0.752 | 2.089 | 1.761 |
| *H’_2_* | 2.296 | 1.659 |  | 2.144 | 1.320 | 1.095 | 0.752 | 2.074 | 1.572 |

^1^HLC/Jan-Dec. ^2,5^CO_2_ baited CDC traps/Jul-Aug. ^4^DCD backpack aspirator, MMT traps/Aug-Nov. ^6^Larval survey/Jan-Dec. ^3,7,8^Larval survey, Ovi traps, BG-Sentinel, BDV tent trap/Oct-Nov, Feb-Mar.

**Reviewer #3**

*22-23: This line is overly general. I suggest making your first sentence more informative about your specific study subject.*

**Response:**

As suggested, we modified the sentence to be more informative and focused on the study subject.

**Modified text (L22-23):**

Mosquito diversity determines disease risk, as only some species transmit pathogens, requiring species’ assemblages identification.

*31: Provide a brief explanation of what Hill numbers are ; e.g. “We calculated Hill numbers to quantify biodiversity using incidence data…”*

**Response:**

As suggested, we edited the text to clarify the use of Hill numbers and other approaches used in this new manuscript’s version.

**Modified text (L27-29):**

Hill numbers of order *q*=0, *q*=1, and *q*=2, non-binomial GLMs, NMDS, PERMANOVA, and generalized estimating equations were used to analyze the diversity, abundance, and phenology.

*Introduction: The introduction section does not flow logically; I suggest reordering (e.g. start with an explanation of vector-borne diseases and zoonoses, then discuss mosquito diversity, and then the impact of the host community, and finally discuss the unique ecology and background info pertaining to the location.) This is just an example of a way that you could reorder it.*

*Make sure you provide all the necessary info for a reader to fully understand (for example, when you mention aegypti and albimanus, you do not explain that these are well described species because they are known vectors of dengue and malaria.*

*The writing needs to be tightened up; for example, lines 70-76 is very repetitive and could be edited to just one sentence. Also 85-87 is repetitive with 70-72. These are just a couple of examples, but there are similar types of repetitive writing throughout the paper. There are also typos (e.g. 116-118 should be “area” and “mangrove”).*

**Response:**

We really appreciate the reviewer’s comments. To ensure writing quality and clarity, we re-written almost completely the Introduction section. As suggested, we tried to follow a logical order in the new manuscript’s version to enhance its coherence, flow, and direct alignment with the study’s objectives.

Specifically, we have:

1. Streamlined the introduction to focus on the diversity and abundance of mosquitoes, ensuring that all background information is directly relevant to the study.
2. Removed content that was not essential for setting the context of mosquito diversity and vector-borne disease transmission.
3. Reorganized the paragraphs to establish a clearer narrative, beginning with the ecological importance of the study region, followed by the significance of mosquito diversity in disease transmission, and concluding with the rationale for our study.
4. Clarified the knowledge gaps that this study aims to address, ensuring a smooth transition into the research objectives.
5. The manuscript’s final version was sent to MDPI editorial services for language revision and editing, ensuring the quality and clarity required by PLoS NTD.

*121: how did you calculate an average distance from a large rectangle with multiple sampling points?*

**Response:**

To estimate the average distance between our study area and the nearest inhabited areas or buildings, we used five measures (from Google Earth). As the reviewer pointed out, there are multiple sampling points. That’s why, instead of using the perimeter of the study site, we used the approximate center location.

*140: What do ECDC and EFSA stand for?*

**Response:**

These are the acronyms for *European Centre for Disease Prevention and Control*, and the *European Food Safety Authority*. We recognise that the reference call was in a wrong format. We ammended that.

**Modified text (L135-138):**

Individuals’ collection was performed mainly through the human landing collection (HLC) method [28]. The HLC technique is one of the oldest methods to catch mosquitoes and has been widely used for its simplicity in collecting host-seeking adult females [28,29].

*143: Who were the collectors? Authors of the paper, volunteers, or employees? Did collectors collect off of themselves or off of each other? How many went out at a time? Was there a human subjects approval (e.g. IRB in the USA, not sure what the equivalent is in Mexico)?*

**Response:**

All collectors were members of the El Carmen laboratory’s working group. Two of them are authors of the paper, and one was a former bachelor’s student. Each collector walked alone along a separate transect, and all three transects were surveyed simultaneously. As a result, each collector collected samples only from their own body, as they were working independently in different locations at the same time. In the new manuscript’s version we clarified this issue.

**Modified text (L147-155):**

During the sampling, we covered 16 24-hour cycles consisting of six 60 min trapping sessions per cycle separated by four hours each: in the mornings at 05:00 and 09:00, in the afternoons at 13:00 and 17:00, and in the night at 21:00 and 01:00 [31]. In addition, for each sampling session, one collector walked independently at its pass along one of the three sampling transects (Fig. 1) for 60 min to trap mosquito females seeking a host. No speed control was imposed, but each transect had to be entirely walked. Each collector was equipped only with an LED head lamp for secure walking after sunset, one 1 L plastic container for mosquito storage, and a pooter composed of a 30 cm glass tube connected to an 85 cm latex hosepipe with filters at each extreme of the pipe. No UV light or other attractors were used for sampling.

In addition, we also clarified that no specific IRB approval is needed for mosquito field collection in Mexico. However, we requested *post facto* IRB approval to the recently created IRB in our institution. After a careful revision of our protocols, we got a favorable opinion. This was also added to the new manuscript’s version.

**Modified text (140-146):**

According to the Regulations of the General Health Law on Health Research [30], consent for HLC is not required in Mexico. However, all collectors were fully informed about the potential infection risks, and all necessary protective measures were implemented to ensure their well-being and safety during the collection process (use of denim clothes and latex gloves to protect as much skin as possible, IRB approval CEID-SOL-2025). Once the collectors agreed with the working conditions, the sampling began. During the study period, the collectors did not show any infection symptoms.

*151: Can you provide more information about how they walked and collected off of themselves at the same time? Was there any control for how long/fast they walked for?*

**Response:**

We edited the methods description to be clearer.

**Modified text (L159-152):**

In addition, for each sampling session, one collector walked independently at its pass along one of the three sampling transects (Fig. 1) for 60 min to trap mosquito females seeking a host. No speed control was imposed, but each transect had to be entirely walked.

*159: Were mosquitoes trapped near dogs held separately to see if there were differences in species attracted to dogs and humans? If not, then it isn’t quite accurate to say you were looking just at anthropophagous mosquitoes, but rather host-seeking mosquitoes.*

**Response:**

No, mosquitoes trapped near or from dogs were not separated. As the reviewer pointed out, using the word anthropophagous was inaccurate; thus, we changed that word throughout the manuscript and referred to the host-seeking adult females as in the new manuscript’s title.

*163-165: How did you use so many keys? Did you use different keys for different genera? Or what was the approach?*

**Response:**

Yes, we used some keys for most of the species. For example, *Carpenter SJ, LaCasse WJ. Mosquitoes of Noth America. Los Angeles, California: University of California Press; 1955* and *Clark-Gil S, Darsie RF. The mosquitoes of Guatemala, their identification, distribution and bionomics. Mosq Syst. 1983;15: 151–199*, were used to identify most of the species; while *Díaz-Nájera A. Claves para indentificar espeices mexicanas de Mansonia y Psorophora (Díptera: Culicidae). Rev Inst Salubr Enferm Trop. 1965;25: 127–137* and *Arnell JH. Mosquitoes studies (Diptera, Culicidae) XXXIII. A revision of the Scapularis group od Aedes (Ochlerotatus). Contrib Am Entomol Institute. 1976;13: 1–44. 35* were used for specific genera or groups.

*170: “seasonal pattern” isn’t the correct term to use here; I suggest using: “… according to the season of each collection event (DS, RS, NS) ….”*

**Response:**

We edited the text as suggested.

**Modified text (L167-169):**

For diversity analyses, the data were grouped according to season (dry, rainy, and norte) of each collection event and the 24-hour sampling cycle. Species richness was estimated using the function *specnumber* of the R package vegan [37].

*179: I think it would be more appropriate to use a generalized linear mixed model so that you can include site as a random effect; you have pseudo-replication of your data points by transect within your study. This would also allow you to include season and hour into the same model.*

**Response:**

We agree with the reviewer. In the new manuscript’s version we substituted the non-parametric KW test by a negative binomial GLM which was complemented with GEE Permanova and NMDS analyses. All this was properly described in the Methods section.

**Modified text (L187-210):**

To further analyze the effects of season (norte, dry, and rainy) and sampling hour (01:00, 05:00, 09:00, 13:00, 17:00, and 21:00) on the abundance of host-seeking female mosquitoes, we used a Negative Binomial Generalized Linear Model (GLM) implemented with the *glm.nb* function from the MASS package [41] in R. This approach was chosen due to the presence of overdispersion in the count data, which violates the assumptions of a Poisson GLM. The Negative Binomial GLM accounts for overdispersion by introducing a dispersion parameter, making it suitable for modeling mosquito abundance data [42]. We applied a Non-Metric Multidimensional Scaling (NMDS) analysis to test clustering patterns across seasons and time of day. NMDS was used to visually explore and reinforce the results of the Negative Binomial GLM, providing a complementary perspective on mosquito activity trends. This ordination approach allows identification of seasonal and diel peaks of organisms’ activity [43], particularly important for areas where species involved in transmitting diseases such as dengue, Zika, and Chikungunya are present. The NMDS was based on a Bray−Curtis dissimilarity matrix derived from species abundance data. The significance of the clustering patterns was tested through a PERMANOVA test using the function *adonis2* from the R package vegan [37]. Additionally, to assess the effects of season, time of day, and vegetation type on the abundance of the five most dominant mosquito genera in the study area, we employed a Generalized Estimating Equations (GEE) approach using the R package geepack [44] to analyze phenological patterns. The GEE model was selected because it accounts for correlated data (e.g., repeated measures across time and space) and allows for robust inference in the presence of non-independence [45,46]. Unlike traditional parametric tests, GEE does not require strict assumptions about the distribution of the dependent variable, making it a suitable choice for our dataset. Only genera with more than 60 records over the study period were included in the analysis to ensure reliable estimates. All analyses were performed in R version 4.1.1 [54].

*182-184: I think that the this would probably be better to include as part of your discussion, but if you want to keep it as part of your methods/results, then I suggest separating the mention of the literature review into a new paragraph and keeping your statement about R connected to your description of the stats.*

**Response:**

We appreciate the reviewer’s comment. As suggested, we separated the mention of the literature review into an independent paragraph while keeping the connection of the R statement to the paragraph describing statistics.

**Modified text (L207-214):**

… traditional parametric tests, GEE does not require strict assumptions about the distribution of the dependent variable, making it a suitable choice for our dataset. Only genera with more than 60 records over the study period were included in the analysis to ensure reliable estimates. All analyses were performed in R version 4.1.1 [47].

To better comprehend the contribution of the Isla del Carmen mosquito species to regional diversity, we compared our data with diversity reports in other areas of the Yucatan Peninsula. We compiled the published works with available abundance data [23,48–54], to estimate species diversity and richness in each state/location as described above.

*200-202: You did not mention that you also collected from buildings and plants in your methods. How was this done? Please provide more info about this.*

**Response:**

In the new manuscript’s version we mention that in the methods section.

**Modified text L(135-139):**

Individuals’ collection was performed mainly through the human landing collection (HLC) method [28]. The HLC technique is one of the oldest methods to catch mosquitoes and has been widely used for its simplicity in collecting host-seeking adult females [28,29]. However, some adult mosquitoes were collected from non-human organisms, including dogs, buildings’ windows and walls, and plant branches and leaves.

*Table 1: can you please provide a citation for each mosquito- pathogen association? Why do you include species richness in your title? I would just highlight seasonal and temporal abundance by mosquito species. Also, you wrote “anthropophagous” mosquitoes in the title; does this mean that this does not include any mosquitoes collected on dogs or plants/buildings?*

**Response:**

Yes. In the new manuscript’s version, we provide a reference list to each mosquito-pathogen association. Because of the size of **Table 1** and the limited space, the list is provided as part of the supplementary material and will be found as **S3 Appendix**. It is pointed out in text:

**Modified text** **(L232-235):**

**Table 1. Seasonal and temporal abundance of host-seeking adult female mosquitoes in Isla del Carmen.** Sampling was carried out during a 24-hour cycle (Time) and three sampling seasons: norte (N), dry (D), and rainy (R). The medical and veterinary relevance of mosquito species was determined by the associated pathogens reported in the literature (see references in S3 Appendix). The last three rows show the total abundance by season across all day times.

As suggested, we removed species richness from the **Table 1** title. We also removed the word anthropophagous to include those species trapped in non-human organisms or other surfaces.

*I think it would be helpful to have another column that gives the total by season across all day times.*

**Response:**

We appreciate the reviewer’s comment. To address this concern, we added three rows at the end of **Table 1.** We decided to add rows instead of an extra column because there is not enough space.

*Would it be potentially easier to read if you divide the hours into groups of day, night, and dawn/dusk? The downside of this approach is that readers would have less detail, but you could include the full table in supplemental.*

**Response:**

We appreciate the reviewer’s suggestions. However, we think that in its actual form, Table 1 shows all relevant information about seasonality and time of day in a non-complicated format.

*218-219: it is confusing whether this list is of genera that were or were not present, please clarify.*

**Response:**

We agree that this paragraph is confusing and thank the reviewer for its observations. To fix this issue we edited the text dropping the species list and keeping only the relevant results.

**Modified text (L244-245):**

All the genera recorded in our study were present in the norte season, while in the dry season, less than 50% of the recorded genera were present.

*Figure 2: it is hard to see the 6 hours since the lines are largely overlapping; it might be better to group by day, night and dusk/dawn. This is the first mention of the three vegetation type groups with acronyms; please define.*

**Response:**

We improved Figure 2 for a better appreciation. As the Reyni diversity profiles are intended to show differences in diversity only when curves do not cross or overlap, we think it is ok to clearly see that diversity is not different across the six daytimes, as it can be seen seasonally. As the reviewer pointed out, in the earlier manuscript’s version we did not define clearly the vegetation type’s acronyms. In this version we fixed that mistake as follows:

**Modified text (L131-135):**

The sampling included three collectors that used pooters (oral aspirators) to trap live-flying adult female mosquitoes on three sampling transects established in 1) a vegetation mixture of dominant mangrove and semideciduous forest (MGSM), 2) a mixture of dominant semideciduous and mangrove forest (SMMG), or 3) a semideciduous forest modified by buildings (BLDSM, Fig. 1).

**Improved Figure 2:**

*I don’t understand what the Renyi diversity profile is or how it was created; can you please provide more information?*

**Response:**

Of course. In the new manuscript’s version, we described the Renyi diversity profiles more accurately:

**Modified text (L175-186):**

To visualize the diversity patterns of the mosquito community, we calculated the Rényi diversity profiles (α), which provide a comprehensive view of species diversity by incorporating multiple diversity indices into a single framework through the function renyicomp of the BiodivesityR package [40]. When α = 0, the Rényi diversity corresponds to species richness; when α = 1, it approximates the Shannon index; and when α = 2, it is equivalent to the Simpson index, which emphasizes species dominance. As α increases, the diversity becomes more influenced by the most abundant species, allowing for a comparison of evenness across communities. We generated Rényi diversity profiles for each sampling season, hour, and vegetation type to compare mosquito assemblages. The profiles were interpreted by assessing how diversity changed across different α values, with curves that remained above others indicating consistently higher diversity across all scales.

*247: How was the diversity analysis conducted?*

**Response:**

In this new version, we described more accurately the method used to conduct our analyses.

**Modified text (L170-175):**

To evaluate the sampling coverage during different seasons and at different times of the 24-hour cycle, we generated accumulation diversity curves. After the conversion of the abundance data to incidence data, we generated three estimates of Hill numbers of order q: 1) species richness (q = 0); 2) Shannon diversity, as the exponential of Shannon entropy or common species (q = 1); and 3) Simpson diversity, calculated as the inverse of the Simpson concentration or dominant species (q = 2), using the functions *iNEXT* and *estimateD* in the R package iNEXT, [38,39]

*253-254: Significance and non-overlapping of confidence intervals are not synonymous in most analyses; if this is different for Hill numbers, please provide a reference*

**Response:**

As suggested, we added the proper references *(Hsieh TC, Ma KH, Chao A. iNEXT: Interpolation and extrapolation for species diversity. 2022; Chao A, Gotelli NJ, Hsieh TC, Sander EL, Ma KH, Colwell RK, et al. Rarefaction and extrapolation with Hill numbers: a framework for sampling and estimation in species diversity studies. Ecol Monogr. 2014;84: 45–67. doi:10.1890/13-0133.1*) to the text **(L281-282)**.

*255: Not clear according to what test?*

**Response:**

We edited the text to be clearer.

**Modified text (282-284):**

According to the Hill numbers, for species richness (*q* = 0), the differences between the rainy and norte seasons were not clear, even when we extrapolated our sampling effort twice (Fig. 3).

*Figure 4: numbers overlap the points, making it hard to read.*

Response:

We re-made Fig.4 to be clearer and more readable.

**New figure 4:**

*Table 3: I believe that it would be better to discuss comparisons between your study and other previous studies in the discussion, but if you decide to keep this table, please add references for each of the other locations.*

**Response:**

We appreciate the reviewer’s comment. Yes, we think that it is valuable to keep the former Table 3 (now Table 4). As the reviewer accurately suggested, we added the references to each location in the title.

**Modified text (L310-317):**

**Table 4. Comparison of female mosquito species richness (*S_r_*) and diversity of Isla del Carmen and other localities in the Yucatan Peninsula.** Parameters were estimated from the data in this study and in the earlier published works [23,47−49,54]. The estimated Shannon−Wiener diversity index (*H’*) by locality included (*H’_1_*) and excluded (*H’_2_*) *Ae. taeniorhynchus* data to show the effect of this eudominant species on the community ecological signal*.* Capture strategy/months are indicated below table with superscripts.

*Did the other locations have a eudominant species other than Ae. taeniorhynchus?*

**Response:**

According to the reviewed articles, although other localities also have *Ae. taeniorhynchus* presence, the populations appear to be not as big as the Isla del Carmen population. No one of the reviewed articles reported eudominant species.

*289: I suggest providing more info about the other papers; e.g. what kind of collection method did they use? Which seasons did they sample in?*

**Response:**

We thank the reviewer’s suggestion. In the new manuscript’s version, the former Table 3 (from the old version) became Table 4. In addition to the added references, in this new version, we also provide information about the diverse capture methods used in earlier surveys, as well as the periods when the catches were performed.

**Modified text (L310-317):**

**Table 4. Comparison of female mosquito species richness (*S_r_*) and diversity of Isla del Carmen and other localities in the Yucatan Peninsula.** Parameters were estimated from the data in this study and in the earlier published works [23,47−49,54]. The estimated Shannon−Wiener diversity index (*H’*) by locality included (*H’_1_*) and excluded (*H’_2_*) *Ae. taeniorhynchus* data to show the effect of this eudominant species on the community ecological signal*.* Capture strategy/months are indicated below table with superscripts.

| Parameter | Isla del Carmen^1^ | Candelaria^2^ |  | Celestún^3^ | Mérida^4^ | Palizada^5^ | Campeche^6^ | Calakmul^7^ | Sian kaan^8^ |
| --- | --- | --- | --- | --- | --- | --- | --- | --- | --- |
| *S_r_* | 26 | 23 |  | 33 | 19 | 11 | 6 | 30 | 26 |
| *H’_1_* | 0.447 | 1.607 |  | 1.629 | 0.751 | 1.377 | 0.752 | 2.089 | 1.761 |
| *H’_2_* | 2.296 | 1.659 |  | 2.144 | 1.320 | 1.095 | 0.752 | 2.074 | 1.572 |

^1^HLC/Jan-Dec. ^2,5^CO_2_ baited CDC traps/Jul-Aug. ^4^DCD backpack aspirator, MMT traps/Aug-Nov. ^6^Larval survey/Jan-Dec. ^3,7,8^Larval survey, Ovi traps, BG-Sentinel, BDV tent trap/Oct-Nov, Feb-Mar, Jun-Jul, Apr-May.

*295: You almost never use this acronym in the paper; I suggest not using it and instead writing it out. I forgot what it was and had to go looking for the definition in the intro.*

**Response:**

We agree with the reviewer’s opinion. As suggested, we dropped the acronym SGoM and instead we used southern Gulf of Mexico throughout the paper (specifically **L64, 357**).

*302: where?*

**Response:**

As requested by the reviewer, we specified the study place of Romero-Vega et al. (2023).

**Modified text (L360-361):**

This result is similar to that of Romero-Vega et al. [59], who reported that in Costa Rica mosquito diversity is greater in the wet season than in the dry season.

*321-323: you may want to remind readers what the temps were for each season here so reader can compare to the ranges you just presented.*

**Response:**

We appreciate the reviewer’s suggestions. As requested, we added information about the environmental features of the study area.

**Modified text:**

**(L362-364)** According to Guerra-Santos and Khal [27], the norte and rainy seasons in our study area have very similar monthly rainfall values (range norte season: 19-207 mm, range rainy season: 92-228mm).

**(L379-382)** During the norte season, the average temperature in our study area was about 2.5 °C lower than in the rainy and dry seasons (25.64 °C, 28.32 °C, and 28.23 °C, respectively) [67], which might have favored increased mosquito abundance.

*329: what do you mean by between? You only sampled at 5 and 9 am, so you don’t have data in between, right?*

Response:

The reviewer’s observation is right. We do not sampled between 05:00 and 09:00 and we do no have data. To be accurate we edited the text.

**Modified text (L392-393):**

… we observed diurnal activity in several species, including *An. crucians*, *An. gabaldoni*, and *An. vestitipennis*, which were inactive only at sunrise and 09:00.

*333: dusk is just during the time when the sun is setting, not the whole night.*

*Also, can you comment whether these diurnal mosquitoes were also observed during the day?*

**Response:**

As requested we added information about the diurnal activity of the species mentioned.

**Modified text:**

**(L391-393)** …we observed diurnal activity in several species, including *An. crucians*, *An. gabaldoni*, and *An. vestitipennis*, which were inactive only at sunrise and 09:00.

**(L394-397)** In our study, typically diurnal species such as *Ae. angustivittatus*, *Ae. scapularis*, *Ae. serratus*, *Ae. aegypti*, and *Ps. ferox* were observed at 09:00, 13:00 and 17:00, but also at 21:00, 01:00 and 05:00, suggesting behavioral flexibility.

*What about other observations of aegypti and other species feeding at night in other publications?*

**Response:**

We added information about studies on the nocturnal activity of *Ae. aegypti*. There is no information on this issue for the other species mentioned in our discussion. We pointed out that more studies are needed to cover this gap.

**Added text (L397-401):**

Recent studies indicate that artificial light at night may disrupt the mosquitoes natural rhythms, leading to increased nocturnal host-seeking activity, particularly in urban and peri-urban environments [73,74], demonstrating that this shift is not incidental but actively stimulated by artificial light exposure [73–75]. However, further research is needed to investigate similar responses of the species recorded in this study to light pollution.

*340-342: I don’t understand this statement; how is the presence of collectors any different than the presence of any human?*

**Response:**

We apologize for the confusing sentence. We mean that the collection method employed (HLC) could have biased the presence of diurnal species. To avoid confusion, we edited the text

**Modified text (L403-408):**

The co-occurrence of diurnal and nocturnal species during crepuscular periods highlights the potential for temporal niche overlap, particularly under transitional (natural or artificial) light conditions [31]. However, these patterns may have been influenced by the presence of collectors [60,73], underscoring the need for future studies to employ alternative sampling methods to minimize bias and better understand the drivers of mosquito activity.

*345: I would assume this is an overestimate of coverage given that the collection method is biased towards mosquitoes that feed on humans, so there are potentially even more mosquito species that weren’t captured because of the HLC bias. I think you should acknowledge this.*

**Response:**

We agree with the reviewer’s comment. We edited the text to acknowledge that there are likely more species in the mosquito assemblage of Isla del Carmen.

**Modified text (L409-413):**

Despite achieving a sampling coverage of 81–94%, our analyses indicated the presence of several undetected species, likely because of the implemented HCL technique. This result suggests that additional sampling effort and complementary collection methods may be necessary to capture rare or elusive species, particularly given the high prevalence of singletons commonly observed in entomological surveys [78].

*363: which species? This is not specific enough to be informative.*

Response:

As requested, we specified the species we refer to.

**Modified text (L431-433):**

These conditions favor *Ae. taeniorhynchus* over other species requiring freshwater for breeding, such as *Ae. aegypti* [80] *Culex coronator* [81], *An. crusians, An. Vestitipennis*, and *An. gabaldoni* [82].

*371: please state which pathogens here in the discussion ( and cite literature that demonstrates role as vector)*

**Response:**

We added the missing information. References to mosquito-pathogen association were stored as supplemental material S3 Appendix.

**Modified text (L436-439):**

Its abundance poses significant health risks, as it is implicated in the transmission of several pathogens, such as VEEV, EEEV, WNV, and *Dirofilaria immitis*, affecting humans and animals (see Table 1, S4 File, and references in S3 Appendix).

*373: It is also a major nuisance mosquito, which can have public health impacts by reducing time spent outdoors.*

**Response:**

We agree with the reviewer’s comment. The health implications are pointed out in the new manuscript’s version **(L439-440)**.

376: “It is important to…” should be removed here and other places where it is used as an introduction to something you are stating; we can assume anything that you are highlighting is important!

**Response:**

We appreciate the reviewer’s comment. As suggested we removed “It is important to” from the sentence.

**Modified text (L441-444):**

Despite being in a semi-conserved area, Ae. aegypti, the sixth most abundant species in our study, was present in the study site, likely due to habitat modifications from nearby human settlements (about 450 m apart). This species is highly adaptable, exploiting both artificial water containers [80] and natural breeding sites, such as tree holes during the rainy season [84].

*386-387: how does it suggest this? I don’t think that this necessarily provides any indication of whether sites were natural or artificial. Please clarify.*

**Response:**

We edited the text to avoid confusion.

**Modified text (L441-446):**

Despite being in a semi-conserved area, *Ae. aegypti*, the sixth most abundant species in our study, was present in the study site, likely due to habitat modifications from nearby human settlements (about 450 m apart). This species is highly adaptable, exploiting both artificial water containers [80] and natural breeding sites, such as tree holes during the rainy season [84]. Its activity throughout all sampling hours, influenced by prey presence and artificial light [73,74], underscores its potential as a vector of regional significant diseases such as dengue and Zika.

*380: can you provide details regarding how far from human residences the Ae. aegypti were collected please? This would help to understand whether the mosquitoes were breeding in the forested habitat or potentially flew from nearby houses.*

**Response:**

Of course. As requested, we added details on the distance of human settlements from the study area **(L443)**.

*Careful to make sure all mentions of Ae. aegypti are written properly with correct capitalization, spelling, and italics.*

**Response:**

We appreciate the reviewer’s observation. We revised the manuscript to ensure that all species names are correctly written.

*401: I don’t understand this sentence; do you mean that only one of all the YP studies did not report the presence of Haemagogus species? Or did they find more/fewer species? Please clarify. – After looking at the reference, I see that the Carpio-Orantes paper is a review paper; this was unclear in the first reading of this paragraph. I suggest rewording.*

**Response:**

In the new manuscript’s version, we changed completely the paragraph about *Haemagogus* species.

**Modified text (L451-455):**

Additionally, we documented *Haemagogus regalis*, which, while not directly implicated in pathogen transmission, deserves monitoring due to the presence of wild primate populations and the potential for wild-type yellow fever virus (YFV) spillover [90], since other *Haemagogus* species are involved in the transmission of sylvatic YFV type [91].

*412: which two species?*

**Response:**

Unfortunately, we do not have an answer to this question. As we pointed out in text, there were two species that were determined only at genus level as *Culex* spp.

**Modified text (L456-459):**

Five *Culex* species were identified in Isla del Carmen, with two remaining unidentified due to morphological limitations.

*445: In English, mosquito exclusively refers to the Culicidae family; suggest rewording.*

**Response:**

In the new manuscript’s version, we present a completely restructured conclusion. Thus, the sentence the reviewer refers to was deleted.

**Modified text (L470-472):**

This study provides the first comprehensive analysis of the 24-hour activity patterns and seasonal dynamics of mosquito populations in Isla del Carmen, a region within the Terminos Lagoon area of the Yucatan Peninsula.

*450-451: statements such as this one should have a citation.*

**Response:**

We agree with the reviewer’s opinion. As the discussion changed, the referred sentence is no more in the text.

*453: It doesn’t really standout; it seems pretty average based on the data from other locations.*

**Response:**

We appreciate the reviewer’s comments and agree. We changed this part of the Conclusion to be more accurate.

**Modified text (L470-474):**

This study provides the first comprehensive analysis of the 24-hour activity patterns and seasonal dynamics of mosquito populations in Isla del Carmen, a region within the Terminos Lagoon area of the Yucatan Peninsula. Our findings reveal similar species richness to other southeastern Gulf of Mexico locations, with *Aedes taeniorhynchus* dominating the mosquito community due to its adaptation to coastal habitats and migratory behavior [83].

*459: Dengue should be lowercase*

**Response:**

We changed from Dengue to dengue.

*470: Should be “funding”*

**Response:**

We appreciate this observation and correct the spelling.

*473: According to PLoS NTD author guidelines: “All data and related metadata underlying the findings reported in a submitted manuscript should be deposited in an appropriate public repository”.*

**Response:**

We appreciate this observation. To be in line with the PLoS NTD author guidelines, we deposited the data associated to this work in the public repository: <https://doi.org/10.5281/zenodo.14633234>

*References: Make sure species names are italicized in references.*

**Response:**

We carefully checked species names to be italicized in references.

*Discussion: it would be good to mention somewhere that you only conducted this study for one year and that most seasonal pattern studies should be conducted over at least 2 years. I understand that time/budget may have been prohibitive; this data is definitely still worth publishing, but this limitation should be noted.*

**Response:**

We agree with the reviewer. As suggested, at the end of the Discussion, we added a final paragraph to note the limitations of the study’s scope.

**Added text (L469-472):**

Although this study was conducted over one instead of two years, as most seasonal pattern studies, it represents the first research in this area in 17 years and documented about twice the number of mosquito species previously recorded, providing valuable baseline data of ecological and public health concerns.
