# Supplementary material for: Abundance and diversity of host-seeking adult female mosquitoes in a coastal ecosystem in southern Mexico
Source: PLoS Negl Trop Dis. 2025 Jun 9;19(6):e0012316. doi: 10.1371/journal.pntd.0012316 (PMC12173415; doi:10.1371/journal.pntd.0012316)
Supplement: Attachment — Submitted filename: Response_to_reviewers_R2.docx [file pntd.0012316.s007.docx]

**Dear Editors of Plos Neglected Tropical Diseases,**

We are thankful for the revision made to our manuscript. We carefully revised and attended to each comment and recommendation from the reviewers to improve our work and increase the odds of being accepted for publication in the journal PLOS Neglected Tropical Diseases.

Below, you will find a response to each comment and the changes made to the original manuscript.

**Reviewer #1**

Reviewer #1: *Authors did a good job by using all reviewers' comments.*

**Response**: We are very thankful with Reviewer #1 for its useful comments and suggestions to improve our work.

**Reviewer #2**

Reviewer #2: *This manuscript examines mosquito diversity on Isla del Carmen over a period of one year. This revised manuscript accomplishes the goals noted in the abstract and needs only minor revisions. Line specific comments are noted below.*

*L23-24: A difficult sentence that could be revised.*

**Response**: Thank you for your feedback. The sentence in question ("L23-24: Mosquito diversity determines disease risk, as only some species transmit pathogens, requiring species’ assemblages identification.") is intentionally concise to emphasize the relationship between mosquito diversity, disease risk, and the necessity of species-level identification. However, we acknowledge that it may appear dense. To improve clarity without losing technical precision, we rewrote the sentence as follows:

L23-24: Mosquito diversity influences disease risk because only certain species transmit pathogens, making the identification of species assemblages essential.

*L49: We examined host-seeking adult female mosquito diversity, abundance....*

**Response**: We edited the sentence as Reviewer #2 suggested.

Modified text

L48-50: We examined host-seeking adult female mosquito diversity, abundance, and activity patterns in southern Mexico’s semi-conserved coastal area of Isla del Carmen.

*L57: Suggest dropping "despite it's dominance."*

**Response**: We modified the sentence as suggested.

Modified text

L56-57: Other medically significant species were recorded, highlighting the potential health risks for humans and wildlife.

*L114: Is this GPS location the centroid of the 10 hectares? Based on the figure, the sampling area is a bounded box so consider providing the GPS locations of the corners of the 10-hectare site.*

**Response**: Reviewer #2 is right. We provided the 10-ha study site’s centroid. As suggested, in the new manuscript’s version, instead of the centroid, we provide the coordinates of polygon’s vertexes.

Modified text

L397-398: The sampling site (vertexes: 18.6517° N -91.7591° W; 18.6507° N -91.7604° W; 18.6555° N -91.7641° W; 18.6567° N -91.7626° W, Fig. 1) is a ten-hectare area,…

*L140-145: Clear statement of IRB approval for study and stated law for HLCs.*

**Response**: We appreciate this comment. Thank you.

*L164: Is there an accession/collection number for this deposit?*

**Response**: No, yet there is no accession/collection number. Our mosquito species collection is in the early stages of formalization. We hope that this year we can have accession numbers for all our records.

*L341-347: This paragraph is not an experimental result so should not be in this section of the manuscript. It is quite appropriate as a part of the discussion.*

**Response**: We agree with the reviewer’s comment. Accordingly, we moved the paragraph to Discussion section, specifically to the Medical significance part.

Modified text

L306-3011: **[Discussion]**

**Medical significance of the mosquito species in Isla del Carmen**

In total, 84% of the mosquito species recorded in this study are recognized vectors of at least 35 pathogen agents, including arboviruses, flaviviruses, and protozoans, which have significant implications for human, domestic animal, and wildlife health (Table 1, S3 Appendix). Among these pathogens are West Nile virus, Rift Valley fever virus, Eastern and Western equine encephalitis viruses, *Dirofilaria immitis*, *Dermatobia hominis*, dengue virus, Zika virus, and Chikungunya virus (Table 1, S4 File).

The species richness in Isla del Carmen was comparable to other regions of the Yucatan Peninsula…

*L366: Suggest immature lifestages rather than Immature mosquito phases.*

**Response**: We modified the sentence as suggested.

Modified text

L242-244: During the norte season, the less abundant rainfall helps immature life stages have more favorable breeding site conditions for developing and increasing the number of emerging adults…

*L406-408: I am glad to see this limitation stated. HLCs are going to be very biased toward species willing to feed on humans. Many Culex and Uranotaenia will not be sampled by HLCs.*

*I suggest that changing collection methods will not probably assist in understanding the drivers of activity but would help to better understand the full scope of mosquito diversity.*

**Response**: We thank the reviewer’s comments and agree with the suggestion. Accordingly, we modified the sentence.

Modified text

L283-285: However, these patterns may have been influenced by the presence of collectors [60,73], underscoring the need for future studies to employ alternative sampling methods to minimize bias and better understand the full scope of mosquito diversity.

*L415-417: Do you think the choice of HLC accounts for the relatively low percentages? What sampling methods were used in these other studies?*

**Response**: Yes, the HCL may have influenced the numbers. Also, the species richness could be higher by sampling other habitat types within Isla del Carmen and using different traps. Accordingly, we modified the paragraph.

Modified text

L291-298: Isla del Carmen harbors a significant proportion of the mosquito diversity reported for the broader region. Specifically, our findings account for 44% of the species documented in the state of Campeche [23,48,49], 35% of those reported in Yucatan [53,54], and approximately 30% of the species recorded in Quintana Roo [48,49,52]. Furthermore, the species richness observed in our study represents approximately 29% of the mosquito diversity recorded across the Yucatan Peninsula [11]. These differences could be reduced by sampling additional habitat types (marshes, coastal dunes, urbanized areas) within Isla del Carmen, and including collection methods mentioned in Table 4 (for example, CO_2_-baited traps).

*Sankey diagram: Species names in left column should be italicized. This should be a pretty easy mod to your R code.*

**Response**: As suggested, we modified the Sankey diagram (supplementary material S4). The new chart shows mosquito species names in italics.
