# Supplementary material for: Abundance and diversity of host-seeking adult female mosquitoes in a coastal ecosystem in southern Mexico
Source: PLoS Negl Trop Dis. 2025 Jun 9;19(6):e0012316. doi: 10.1371/journal.pntd.0012316 (PMC12173415; doi:10.1371/journal.pntd.0012316)
Supplement: Attachment — Submitted filename: Response_to_Editors_Comments.docx [file pntd.0012316.s009.docx]

Dear editor,

We revised all your valuable comments and suggestions and conducted the proper editions of the text. Accordingly, our work now meets all the quality requirements of Plos NTD to enhance its odds of being accepted for publication. Below, you will find a response to each comment/suggestion and the corresponding text editions carried out, indicated with its line number to easy location.

# Response to Editor’s comments

# Abstract

*L27-28: Suggest to list the climate seasons: norte, rainy and dry*

**Response:** As suggested, we edited the sentence.

**Modified text:**

L27-28: cycles in mangrove and low-semideciduous forest patches across three climate seasons: norte, rainy and dry.

*L30: “… analyze mosquito diversity, abundance and phenology.”*

**Response:** We edited the sentence according to the Editor’s suggestion.

**Modified text:**

L30-31: were used to analyze mosquito diversity, abundance, and phenology.

*L35: Suggest to list the five genera*

**Response:** Following the Editor’s suggestion, we listed the top five genera.

**Modified text:**

L35-36: The top five genera, *Aedes*, *Psorophora*, *Mansonia*, *Culex* and *Anopheles*, exhibited distinct phenological patterns,…

*L36: Suggest to include years: September 2019 and February 2020*

**Response:** As suggested, we include the years to the text.

**Modified text:**

L37: with abundance peaking between September 2019 and February 2020.

*L38: Suggest to include a brief description (moderate rainfall, cooler temperatures).*

**Response:** According to the Editor’s suggestion, we modified the sentence.

**Modified text:**

L39-40: Our findings highlight the norte season, when cooler temperatures and moderate rainfall are present, as a critical period for mosquito activity,…

*L41: Suggest to replace to coastal areas in southern Mexico or Yucatan Peninsula.*

**Response:** We edited the text according to the Editor’s suggestion.

**Modified text:**

L42-43: This study provides valuable insights into mosquito community dynamics and their implications for public health in coastal areas of southern Mexico.

# Author summary

*L50: Suggest to include sampling method (human landing collection)*

**Response:** We modified the text according to the Editor’s suggestion.

**Modified text:**

L52-53: Human landing collection sampling was conducted monthly in mangrove and semideciduous forest…

*L54: Suggest to indicate percentage or number. Several is too vague.*

**Response:** We agree with the Editor, accordingly, we edited the text.

**Modified text:**

L54-55: Among the 26 recorded species, 84% are known vectors of diseases

*L56: Suggest to replace to “medically important mosquito species”*

**Response:** We edited text according to Edito’s suggestion.

**Modified text:**

L59-59: Other medically important mosquito species were recorded…
